# Straightforward Synthesis of Superparamagnetic Fe_3_O_4_‑Carbon Dot Nanohybrids for Environmental Remediation: A Model Study Using Congo Red

**DOI:** 10.1021/acsomega.6c08673

**Published:** 2026-10-28

**Authors:** Arthur Hegermann Ferreira, Pedro Lana Gastelois, Leandro Vinícius Alves Gurgel, Fabiano Vargas Pereira

**Affiliations:** † Department of Chemistry, Universidade Federal de Minas Gerais, Av. Antônio Carlos, 6627, Pampulha, Belo Horizonte CEP 31270-901, Brazil; ‡ 54530 Centro de Desenvolvimento da Tecnologia Nuclear, Av. Antônio Carlos, 6627, Pampulha, Belo Horizonte CEP 31270-901, Brazil; § Department of Chemistry, Universidade Federal de Ouro Preto, Campus Morro do Cruzeiro, Rua Quatro, 786, Bauxita, Ouro Preto CEP 35402-136, Brazil

## Abstract

Carbon dots (CDs) have attracted increasing interest as carbon-based nanomaterials due to their tunable surface chemistry, colloidal stability and low toxicity. Among them, magnetic carbon dots (MCDs) constitute a promising multifunctional subclass. In this work, magnetite-based magnetic carbon dots (Fe_3_O_4_-MCDs) were synthesized through a straightforward route using only ammonium ferric citrate and ethylenediamine as precursors. TEM revealed quasi-spherical Fe_3_O_4_-MCD nanohybrids with an average particle size of 12 ± 3 nm. XPS depth profiling employing Ar^+^ ion sputtering showed that these nanoparticles possess a magnetite-rich interior and a functionalized carbon-rich outer region. Furthermore, these nanoparticles self-assembled into hierarchical architectures with interparticle voids, resulting in a porous network with a specific surface area of 107 m^2^ g^–1^ and a pore volume of 0.431 cm^3^ g^–1^. In addition, the negligible coercivity and remanence are consistent with superparamagnetic-like behavior at room temperature, with a saturation magnetization of ∼60 emu g^–1^, facilitating rapid magnetic separation and redispersion. The Fe_3_O_4_-MCDs were evaluated for the adsorption of Congo red as a model pollutant, achieving an adsorption capacity of 121.6 mg g^–1^. Nonlinear kinetic analysis showed that adsorption followed the mixed 1,2-order model, while the Sips isotherm best described the equilibrium data. Overall, the results demonstrate that the Fe_3_O_4_-MCDs combine straightforward synthesis, high saturation magnetization, superparamagnetic behavior, and pollutant adsorption performance. These properties arise from their carbon/magnetite nanoarchitecture and engineered interfacial chemistry, promoting interactions with pollutants and highlighting their potential for multifunctional contaminant removal.

## Graphical abstract

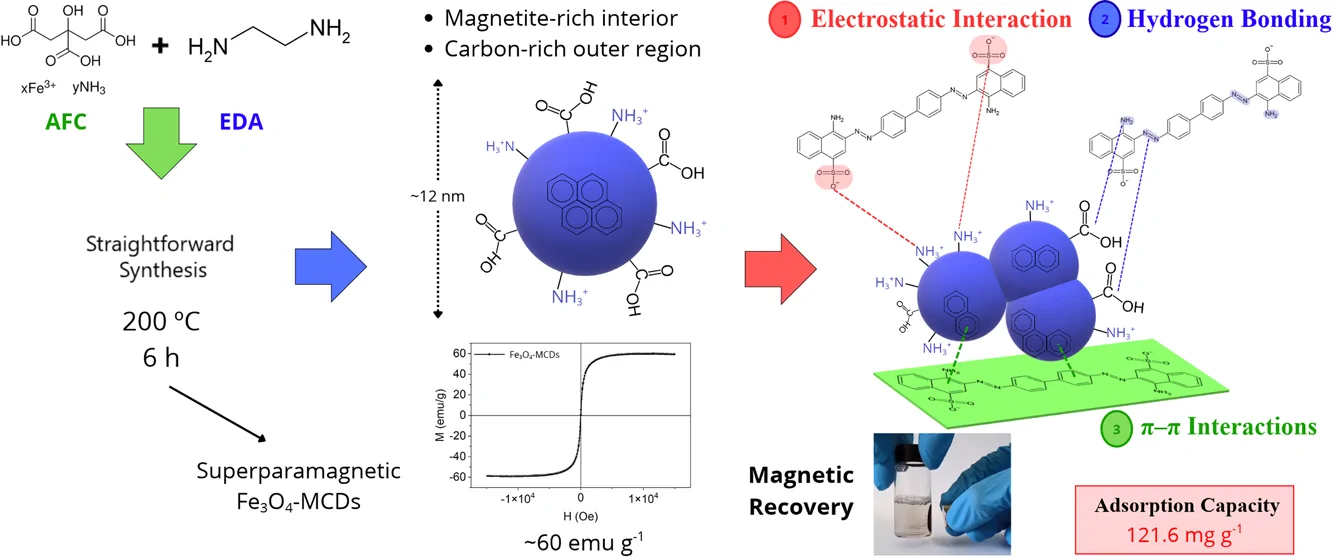

## 1 Introduction

Among the carbon nanomaterials investigated over the past decades, carbon dots (CDs) are notable for the intricate relationship between structure, composition, and properties, since their apparent simplicity as 0D systems contrasts with a complexity that remains only partially understood.^1,2^ To some extent, this complexity arises from the fact that CDs do not constitute a single material but rather a class of nanocarbon materials with tunable properties, high functional versatility, and an expanding range of applications across diverse fields.^3,4^

This family of nanocarbons consists of nanoscale carbon-based entities (typically 2–10 nm) with mixed sp^2^/sp^3^ hybridization that can form stable colloidal dispersions and exhibit strong and tunable photoluminescence.^5,6^ They generally display colloidal stability in water, low toxicity, and remarkable chemical robustness, while their properties can be tailored through variations in both core structure and surface chemistry.^7,8^ Moreover, their properties can be readily tailored through the choice of precursors, synthesis conditions, and surface functionalization strategies, making CDs highly versatile building blocks for the development of multifunctional hybrid nanomaterials.^4,9^

CDs can be synthesized through either top-down or bottom-up approaches.^5,10^ While top-down methods generate nanoscale carbon structures from larger carbonaceous materials, bottom-up strategies rely on the carbonization of molecular precursors and offer greater flexibility in terms of composition, surface chemistry, and functional properties.^3,4^ Among the latter, hydrothermal carbonization has emerged as one of the most used and versatile routes for the preparation of CDs, enabling the production of nanomaterials with diverse structures and surface functionalities.^7,11^ This synthetic versatility has driven the development of hybrid and multifunctional CD-based materials.

In the context of the broad diversity and multifunctionality of CDs, the incorporation of magnetic properties into these materials has enabled the development of magnetic carbon dots (MCDs),^12,13^ a class of hybrid nanomaterials that combines the characteristic features of CDs, including photoluminescence, colloidal stability, and versatile surface chemistry, with the magnetic behavior of inorganic nanoparticles, particularly superparamagnetism.^14,15^ This combination of properties significantly expands the potential applications of CDs in areas such as magnetic resonance imaging,^16,17^ controlled drug delivery,^18,19^ and preconcentration strategies for analytical applications.^13^ In addition, environmental uses of MCDs such as the detection and removal of pollutants also represent promising prospects for these magnetic nanomaterials.^14,20^ However, despite some advances in the area of environmental applications of MCDs, there is still a lack of systems that simultaneously combine facile synthesis, strong adsorption performance, high saturation magnetization, and superparamagnetic behavior. Such a combination can enable efficient and fast magnetic recovery of pollutants under an external magnetic field while reducing the tendency for persistent magnetic aggregation after removal of the external magnetic field. In addition, most reported systems rely on multiple nitrogen-rich synthetic precursors (e.g., ethylenediamine derivatives such as DETA or TETA),^14,21^ increasing cost and limiting scalability, while the use of low-cost and few precursors for MCDs remains underexplored.

In particular, although superparamagnetic behavior is highly desirable for environmental remediation because it enables efficient magnetic separation while minimizing particle aggregation after removal of the external magnetic field, some reported systems still exhibit relatively low saturation magnetization values (emu g^–1^), which could limit the speed and efficiency of magnetic recovery in practical remediation processes.^22,23^

Thus, in this work, a straightforward synthetic route is proposed for the preparation of a magnetite-based carbon dot nanohybrid (Fe_3_O_4_-MCDs) using only two low-cost reagents. The obtained material was characterized by several techniques, including a detailed XPS sputtering study, which revealed a functionalized carbon-rich surface layer and Fe_3_O_4_-rich inner regions in the nanoparticles. This integrated carbon/Fe_3_O_4_ nanoarchitecture played a key role in the properties achieved by the nanomaterial, with the Fe_3_O_4_ inner regions providing high saturation magnetization and superparamagnetic behavior for rapid and efficient magnetic recovery, while the carbon-rich surface layer provided functional groups that promoted interactions with pollutant molecules and enhanced adsorption performance. The adsorption efficiency of the Fe_3_O_4_-MCDs was evaluated for the adsorption of Congo red dye, used as a model pollutant, showing significant adsorption capacity. A detailed analysis of the adsorption behavior, based on nonlinear kinetic and different isotherm models, combined with textural, morphological, and FTIR analyses of the Fe_3_O_4_-MCD system, provided insights into the adsorption process and the interfacial interactions associated with pollutant removal.

The results show that the Fe_3_O_4_-MCD nanohybrid can constitute a versatile platform for environmental applications, as its surface properties can be tuned to enable specific interactions with various chemical contaminants and pollutants, significantly expanding its potential in this field.

## 2 Experimental Section

### 2.1 Materials

Ammonium ferric citrate (AFC) was obtained from Êxodo Científica (Sumaré, Brazil). Ethylenediamine (EDA) was purchased from Neon (Suzano, Brazil) while Congo red dye was acquired from Imbralab (Ribeirão Preto, Brazil). Sodium hydroxide, hydrochloric acid, and potassium chloride were obtained from FMaia (Belo Horizonte, Brazil), Química Moderna (Santana de Parnaíba, Brazil), and Vetec Química fina LTDA (São Paulo, SP, Brazil), respectively. All reagents were used as received, without any further purification.

### 2.2 Synthesis of Fe_3_O_4_-Magnetic Carbon Dots

A solution of AFC (0.4 g) was prepared in 20 mL of water, followed by the addition of 3 mL of EDA. The mixture was magnetically stirred for several minutes until complete dissolution of the reagents was achieved. The solution volume was then adjusted to 30 mL and transferred to a stainless-steel autoclave lined with a Teflon vessel. The system was heated to 200 °C and maintained at this temperature for 6 h. After the reaction, the autoclave was allowed to cool naturally to room temperature. The obtained material was carefully separated using a magnet and washed three times with isopropanol to remove soluble and weakly associated species, including free CDs and residual organic compounds. The final product was then dried in an air-circulating oven at 60 °C. Approximately 100 mg of dried product was typically obtained per batch, corresponding to a yield of approximately 25%, calculated based on the initial mass of AFC, since EDA was employed in excess as a reducing agent and nitrogen source during the synthesis. The synthesis was repeated in independent batches under the same conditions and showed good reproducibility in terms of product recovery and physicochemical characteristics.

### 2.3 Characterization Methods

UV–vis absorption measurements were performed on a Hitachi U-2010 spectrophotometer. The spectra were obtained using diluted aqueous suspensions placed in a quartz cuvette with a 1 cm optical path length.

Photoluminescence (PL) measurements were performed using a Varian Cary Eclipse fluorescence spectrometer with a 1 cm path length quartz cuvette. Emission spectra were recorded at different excitation wavelengths.

The fluorescence quantum yield (QY) was determined using quinine sulfate in 0.1 mol L^–1^ H_2_SO_4_ as the reference standard. Both the sample and the standard were measured under identical experimental conditions, and the QY values were calculated according to eq 1.^24^

$$\Phi_{MCD}=\Phi_{\mathrm{ST}}\left(\frac{S_{MCD}}{S_{\mathrm{ST}}}\right)\left(\frac{\eta_{MCD}^{2}}{\eta_{\mathrm{ST}}^{2}}\right)$$ 1

where *S* is the slope of the integrated emission intensity versus absorbance plot, MCD refers to the sample, ST to the standard, Φ is the quantum yield, and η is the refractive index, assumed to be 1.33 for the diluted solutions.

Transmission electron microscopy (TEM) was employed to investigate the morphology, particle size and size distribution of Fe_3_O_4_-MCDs. For analysis, dilute dispersions were prepared and a drop of each suspension was deposited onto ultrathin carbon-coated copper grids (≈3 nm film thickness), followed by drying at ambient conditions. The measurements were carried out using an FEI Tecnai G2–20 SuperTwin microscope operated at an accelerating voltage of 200 kV. Scanning electron microscopy (SEM) was performed using an FEI Quanta 200 FEG microscope. Raman spectroscopy measurements were performed using a WITec alpha300 RA spectrometer equipped with a 532 nm excitation laser. Diluted aqueous dispersions were drop-cast onto clean glass substrates and allowed to dry under ambient conditions prior to analysis. Fourier-transform infrared (FTIR) spectra were recorded using a Shimadzu IRSpirit spectrometer. The measurements were performed in the 4000–400 cm^–1^ spectral range using KBr pellets prepared with the powdered samples. XRD analysis was carried out using an Anton Paar XRDynanic-500 diffractometer, with data collected from powdered samples over a 2θ range of 10–80°. Magnetic measurements of Fe_3_O_4_-MCDs were carried out using a vibrating sample magnetometer (VSM, Lake Shore 7400). Magnetization curves were recorded at room temperature as a function of the applied magnetic field. X-ray photoelectron spectroscopy (XPS) measurements were carried out using a UHV system equipped with a Phoibos-150 analyzer (SPECS) and Al Kα radiation (1486.6 eV). Survey and high-resolution spectra were acquired at pass energies of 100 and 30 eV, respectively. Binding energies were calibrated using the C 1s peak at 284.6 eV. Spectral analysis was performed using CasaXPS and KherveFitting software,^25^ employing Shirley background subtraction and Gaussian–Lorentzian line shapes. Depth profiling measurements were performed through successive Ar^+^ sputtering steps (3.0 keV), followed by XPS acquisition after each sputtering cycle. Surface area measurements were obtained from N_2_ adsorption–desorption analyses at 77 K using an Autosorb iQ analyzer (Quantachrome Instruments). Flame atomic absorption spectrometry (FAAS) was used to quantify Fe leaching from the Fe_3_O_4_-MCD nanohybrid under the investigated conditions using a VARIAN AA240FS atomic absorption spectrometer equipped with a flame atomizer. The specific surface area was calculated by the Brunauer–Emmett–Teller (BET) method, the total pore volume by the Gurvich method, and the pore size distribution by the Barrett–Joyner–Halenda (BJH) method using the adsorption branch. Zeta potential measurements were performed using an Anton Paar Litesizer DLS 500. The analyses were carried out on diluted aqueous dispersions at different pH values.

### 2.4 Adsorption Experiments

For the adsorption experiments, Congo red (CR) dye was employed as a model pollutant. The concentration was determined by UV–vis absorption spectroscopy at 509 nm using three analytical calibration curves established over different concentration intervals, namely a high-concentration range of 80–150 mg L^–1^, an intermediate range of 20–60 mg L^–1^, and a low-concentration range from 0.0 to 10 mg L^–1^, in which the first nonzero calibration point was 0.5 mg L^–1^. For the kinetic experiments, approximately 8.0 ± 0.1 mg of previously dried Fe_3_O_4_-MCDs were weighed into 50 mL Erlenmeyer flasks. Then, 25.00 mL of Congo red solutions at concentrations of 40.00, 15.03, 5.65, and 2.10 mg L^–1^ were added to each flask. The pH of the solutions was adjusted using 0.01 mol L^–1^ HCl or NaOH, as required, to determine the conditions for maximum adsorption efficiency. The temperature was maintained at 25 ± 1 °C throughout the experiments. After the addition of the Congo red solution, the adsorbent–adsorbate systems were kept under agitation in an orbital shaker at 420 rpm for different time intervals. At each sampling time, the adsorbent (Fe_3_O_4_-MCD) was magnetically separated, and the resulting supernatant was collected for UV–vis spectroscopic analysis. The residual dye concentration was monitored at regular intervals during the experiment for up to 240 min. The dye removal efficiency (*R*, %) and the adsorption capacity at time *t* (*q*_*t*_, mg g^–1^) were calculated at each sampling interval according to eqs 2 and 3, respectively

2 $$R=\left(\frac{C_{0}-C_{t}}{C_{0}}\right)\times 100$$

3 $$q_{t}=\frac{\left(C_{0}-C_{t}\right)V}{m}$$

where *C*_0_ (mg L^–1^) is the initial concentration of the Congo red solution, *C*_*t*_ (mg L^–1^) is the concentration after an adsorption time *t* (min), *V* (L) is the total volume of the solution, and *m* (g) is the mass of the adsorbent.

Four different kinetic models were evaluated to describe the adsorption of Congo red onto Fe_3_O_4_-MCD nanoparticles: pseudo-first order (PFO),^26^ pseudo-second order (PSO),^27^ mixed 1,2-order (MO) kinetic model,^28^ and intraparticle diffusion model.^29^ The PFO and PSO models are described according to the following equations

$$q_{t}=q_{e}\left(1-e^{-k_{1}t}\right)$$ 4

5 $$q_{t}=\frac{k_{2}q_{e}^{2}t}{1+k_{2}q_{e}t}$$

where *q*_e_ and *q_t_* (mg g^–1^) are the adsorption capacities at equilibrium and time *t* and *k*_1_ (min^–1^) and *k*_2_ (g mg^–1^ min^–1^) are the PFO and PSO rate constants, respectively.

The MO model is described according to the following equations

$$\frac{dq_{t}}{\mathrm{dt}}=\frac{{k_{1}}^{\prime }}{\left(1-f_{2}\right)q_{e}}\left(q_{e}-q_{t}\right)\left(q_{e}-f_{2}q_{t}\right)$$ 6

7 $$f_{2}=\frac{{k_{2}}^{\prime }q_{e}}{{k_{1}}^{\prime }+{k_{2}}^{\prime }q_{e}};f_{1}+f_{2}=1$$

where *f*_1_ and *f*_2_ are the PFO and PSO model contributions and *k*_1_^’^ (min^–1^) and *k*_2_^’^ (g mg^–1^ min^–1^) are the PFO and PSO rate constants in the MO model, respectively. When *f*_2_ = 0 or *f*_2_ = 1, the MO model becomes the pure PFO or PSO model, respectively.

The intraparticle diffusion model is described according to the following equation

$$q_{t}=k_{\mathrm{id}}t^{\left(1/2\right)}+C_{\mathrm{int}}$$ 8

where *k*_id_ (mg g^–1^ min^–1/2^) is related to an intraparticle diffusion rate constant. *C*_int_ (mg g^–1^) is the intercept of the linear fit of *q_t_* versus *t* ^1/2^.When the *q_t_* versus *t* ^1/2^ plots exhibited more than one linear region, the linear regions were fitted to obtain the corresponding slopes and intercepts.

For the isotherm experiments, approximately 8.0 ± 0.1 mg of the adsorbent were again transferred into Erlenmeyer flasks, to which 25.00 mL of Congo red solutions at concentrations of 4, 8, 16, 24, 32, 40, 50, 60, 75, 90, 110, and 125 mg L^–1^ were added in triplicate. Each system was kept under agitation in an orbital shaker at 420 rpm and 25 ± 1 °C. After the equilibrium time of 240 min, the equilibrium adsorption capacity was determined as a function of the equilibrium dye concentration (*C*_e_). Four different isotherm models were evaluated to describe the adsorption equilibrium of Congo red onto Fe_3_O_4_-MCD nanoparticles: the Langmuir,^30^ Freundlich,^31^ Sips,^32^ and Dubinin–Radushkevich models.^33^ The Langmuir model is described according to the following equation

$$q_{e}=\frac{Q_{\max}bC_{e}}{1+bC_{e}}$$ 9

where *C*_e_ (mg L^–1^) is Congo red concentration at equilibrium, *Q*_max_ (mg g^–1^) is the Congo red maximum adsorption capacity, *q*_e_ (mg g^–1^) is the Congo red equilibrium adsorption capacity, and *b* (L mg^–1^) is the Langmuir binding constant.

The Freundlich model is described according to the following equation

$$q_{e}=K_{F}{C_{e}}^{1/n}$$ 10

where *K*_F_ (mg g^–1^)­(L mg^–1^)^1/*n*^ is the Freundlich empirical constant that characterizes the adsorption capacity and *n* (dimensionless) is a model fit parameter.

The Sips model is described according to the following equation

$$q_{e}=\frac{Q_{\max}{\left(bC_{e}\right)}^{1/n}}{1+{\left(bC_{e}\right)}^{1/n}}$$ 11

where *n* is a parameter that can be associated with the heterogeneity of the adsorption system. Therefore, if this parameter is equal to unity, eq ([Disp-formula eq11]) becomes the Langmuir isotherm (eq ([Disp-formula eq9])).

The Dubinin–Radushkevich model is described according to the following equation

$$q_{e}=q_{s}\exp\left(-B\varepsilon^{2}\right)$$ 12

where *q*_s_ (mg g^–1^) is the maximum adsorption capacity of Congo red on Fe_3_O_4_-MCD, *B* (mol^2^ kJ^–2^) is a parameter associated with the adsorption characteristic energy taking as a reference benzene, and *ε* (kJ mol^–1^) is the Polanyi’s potential, calculated using the following equation

13 $$\varepsilon =\mathrm{RT}ln\left(\frac{C_{s}}{C_{e}}\right)$$

where *C*_e_ (mg L^–1^) is the equilibrium concentration of Congo red and *C*_s_ (mg L^–1^) is the saturation concentration of Congo red in solution. *T* (K) is the absolute temperature and *R* (0.008314 kJ K^–1^ mol^–1^) is the gas constant.

The adsorption characteristic energy of the Dubinin–Radushkevich model is calculated using the following equation

14 $$E_{D‐R}=\frac{1}{\sqrt{2B}}$$

where *E*_D‑R_ (kJ mol^–1^) is the characteristic energy parameter obtained from the Dubinin–Radushkevich model.

For desorption and reusability studies, Congo red-loaded Fe_3_O_4_-MCDs were regenerated using 0.01 mol L^–1^ NaOH or KOH solutions. Independent adsorbent samples were subjected to five consecutive adsorption–desorption cycles. In each cycle, adsorption was performed using 25.00 mL of Congo red solution (60.0 mg L^–1^, pH 3) for 90 min at 25 ± 1 °C and 420 rpm. After magnetic separation, the residual dye concentration was determined by UV–vis spectroscopy. Desorption was carried out by contacting the magnetically separated dye-loaded adsorbent with 25.00 mL of the regenerating solution for 120 min at 25 ± 1 °C and 420 rpm. Between cycles, the material was washed with water, reconditioned at pH 3, and kept wet without drying.

Because incomplete desorption may lead to progressive accumulation of Congo red on the adsorbent over successive cycles, a mass-balance correction was applied to determine the effective desorption efficiency. Details of the calculations are provided in the , including the used to determine desorption efficiency and reusability. Reusability was evaluated by comparing the adsorption capacity obtained in each cycle with that of the first cycle.

## 3 Results and Discussion

### 3.1 Optical Properties and Chemical Characterization

In general, the optical properties of conventional CDs (in the absence of a magnetic phase) are reported to be strongly influenced by the chemical nature of the surface functional groups of these nanoparticles.^1,11^ In this context, the optical behavior of the obtained Fe_3_O_4_-MCDs is expected to remain strongly influenced by their surface functional groups. Therefore, aspects related to optical response and surface composition are discussed together in this subsection, enabling a more integrated analysis of the structure–property relationship in these nanostructures.

Figure 1 presents the optical characterization of the Fe_3_O_4_-MCDs. The UV–vis spectrum (Figure 1a) exhibits an absorption band in the UV region (∼230 nm), attributed to π–π* transitions of C=C bonds associated with sp^2^-hybridized domains.^34,35^ A second band centered at ∼330 nm is also observed, which can be associated with n−π* transitions of C=O (or C=N) groups, indicating the presence of surface functional groups.^36^ Additionally, a weak absorption tail extending into the visible region (λ > 400 nm) suggests the presence of lower-energy electronic transitions associated with surface states and defect-related energy levels.^37^

**Figure 1:**
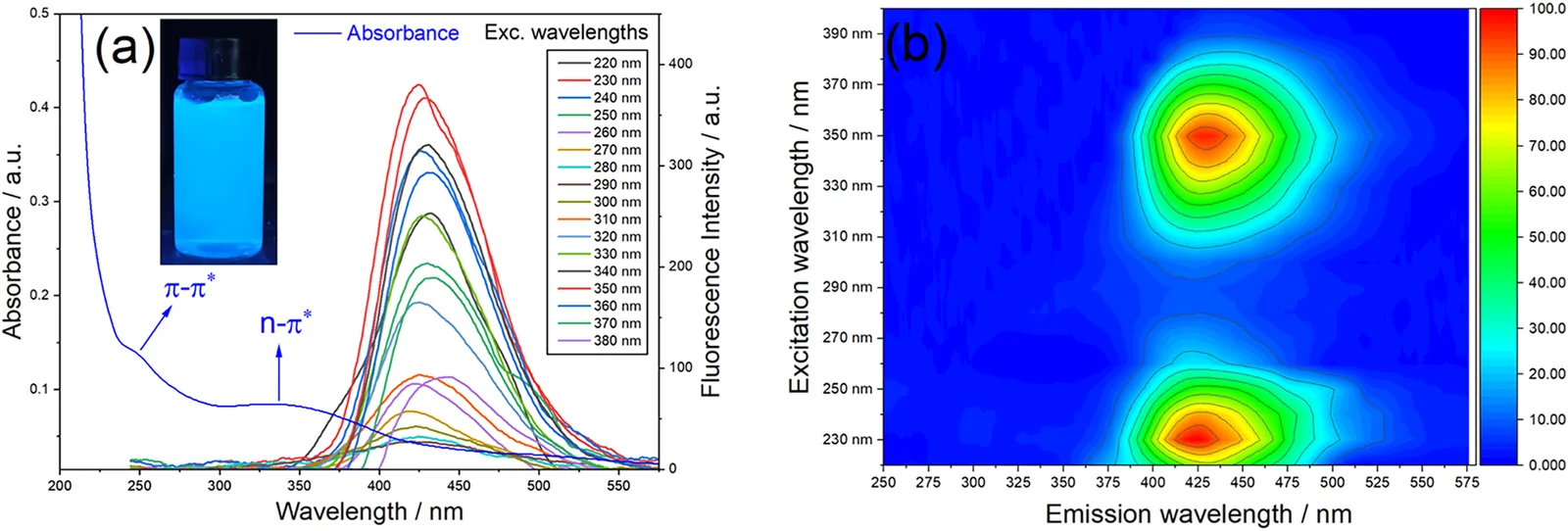
(a) UV–vis absorption spectrum and fluorescence emission spectra of Fe_3_O_4_-MCDs at different excitation wavelengths (as indicated). The inset shows the sample dispersion in water under UV irradiation (365 nm). (b) Two-dimensional fluorescence contour plot of Fe_3_O_4_-MCDs.

The inset in Figure 1a shows the appearance of the Fe_3_O_4_-MCDs dispersion in water under UV irradiation (λ = 365 nm). The sample exhibits a strong blue fluorescence under UV illumination. The fluorescence emission spectra of the Fe_3_O_4_-MCDs (Figure 1a), recorded at excitation wavelengths ranging from 220 to 380 nm in 10 nm intervals, exhibit emission maxima at approximately 430 nm, independent of the excitation wavelength. In conventional CDs, excitation-dependent photoluminescence is commonly attributed to a heterogeneous distribution of surface functional groups, which gives rise to multiple emissive states at different energy levels.^38^ On the other hand, excitation-independent behavior suggests the presence of uniform emissive states, typically associated with a relatively homogeneous size distribution and well-defined core or surface electronic structure.^39^ In the case of the obtained Fe_3_O_4_-MCDs, the observed excitation-independent behavior suggests a uniform size distribution and a relatively homogeneous surface functionalization. The optical characterization of the Fe_3_O_4_-MCDs, based on UV–vis absorption and fluorescence emission, indicates that these nanomaterials exhibit fluorescence behavior similar to that of conventional CDs. This result suggests that the incorporation of the Fe_3_O_4_ phase does not significantly alter the characteristic optical properties of these nanomaterials.

Additionally, an interesting feature of the photoluminescence properties of the Fe_3_O_4_-MCDs was observed. The sample exhibits two emission maxima, one when excited at 230 nm and another upon excitation at 350 nm. These two emission maxima are associated with the excitation of two distinct emissive centers: one related to carbon domains rich in sp^2^ character, excited at 230 nm (higher energy), predominantly via π–π* transitions, and another associated with surface states, excited at 350 nm, involving n−π* transitions arising from functional groups.^34,36^ These two emission maxima can be more clearly observed in the two-dimensional fluorescence map (fluorescence contour plot) shown in Figure 1b.

The fluorescence quantum yield (QY) was determined from the linear correlation between the integrated photoluminescence (PL) intensity and absorbance at different concentrations of the sample and reference. The fluorescence QY was determined to be 49.2%, and the corresponding curves are presented in Figure S1 (Supporting Information). Interestingly, the value obtained for the Fe_3_O_4_-MCDs is comparable to those reported for CDs synthesized from citric acid and ethylenediamine,^40^ again indicating that the incorporation of the magnetic phase does not significantly affect the optical properties of conventional carbon nanoparticles.

The chemical characterization of the Fe_3_O_4_-MCDs was carried out using FTIR and XPS, providing detailed information on the surface functional groups of the carbonaceous fraction and the iron species present. The results obtained are summarized in Figure 2.

**Figure 2:**
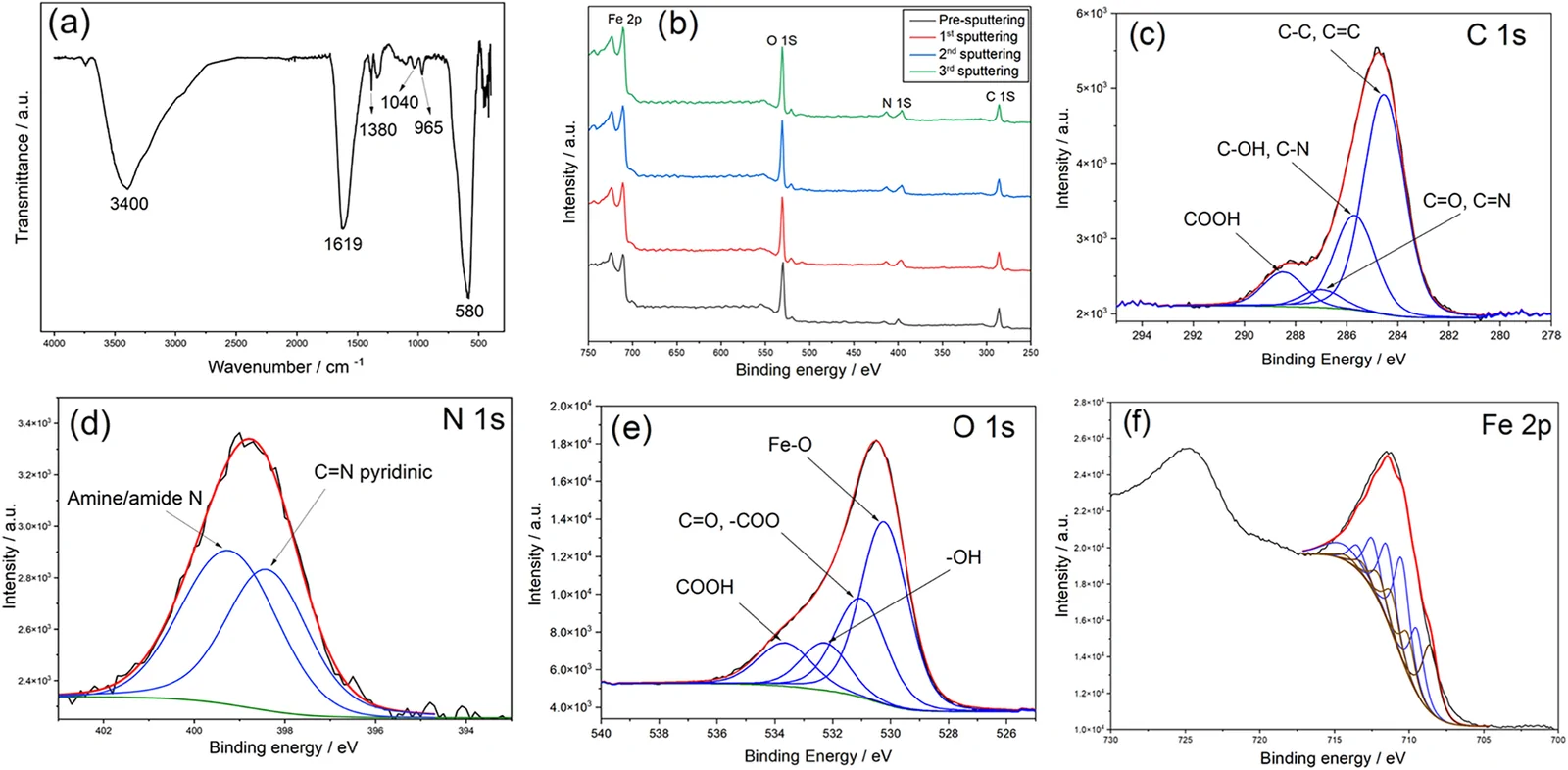
FTIR spectrum (a), XPS survey spectra before and after sputtering (b), and high-resolution XPS spectra of C 1s (c), N 1s (d), O 1s (e), and Fe 2p (f) for the Fe_3_O_4_-MCDs.

The FTIR spectrum shown in Figure 2a exhibits a broad absorption band centered at ∼3400 cm^–1^, which is attributed to overlapping O–H and N–H stretching vibrations associated with hydroxyl (including phenolic), carboxylic, amine, and/or amide surface groups.^41^ A broad band with a maximum at 1619 cm^–1^ (1670–1505 cm^–1^) can be attributed to overlapping contributions, mainly associated with C=C and/or C=N stretching vibrations, related to sp^2^ domains and nitrogen-containing functionalities.^42^ The peak centered at 1380 cm^–1^ can be associated with C–N stretching vibrations and/or symmetric stretching of carboxylate groups (−COO^–^), indicating the presence of oxygen- and/or nitrogen-containing functionalities on the surface.^41^ The band at ∼1040 cm^–1^ is attributed to C–O stretching vibrations associated with oxygen-containing surface groups such as C–OH and/or C–O–C functionalities. The signal observed near ∼965 cm^–1^ may be associated with out-of-plane aromatic C–H bending vibrations from substituted aromatic domains present in the carbon structure.^42,43,44^ Finally, the intense band at ∼580 cm^–1^ is assigned to Fe–O stretching vibrations, providing clear evidence of an iron oxide phase and being consistent with the formation of Fe_3_O_4_.^14,44^

Figure 2b–2f present the XPS analysis of the Fe_3_O_4_-MCDs. To investigate the elemental composition at the surface and within the inner regions of the material, an XPS depth-profiling study was performed using successive Ar^+^ sputtering steps (3.0 keV), with XPS spectra acquired after each step. Figure 2b shows the survey spectra obtained before and after successive sputtering processes, allowing the progressive removal of surface layers and highlighting compositional changes with increasing depth. This approach provides access to subsurface regions, revealing variations in the relative intensities of the elements. The corresponding elemental compositions (atomic percentages) of iron and carbon for each region, before and after the sputtering steps, are summarized in Figure S2. Successive sputtering steps led to a gradual increase in the Fe content and a concomitant decrease in the carbon percentage, consistent with the exposure of Fe-rich inner regions after removal of carbon-rich surface layers. The carbon atomic percentage decreased from 29.3% before sputtering to 22.3% after the third sputtering step, whereas the Fe content increased from 25.4% to 32.2% after the third sputtering step.

In this way, the compositional evolution observed during successive sputtering steps in the XPS depth-profiling analysis supports the formation of magnetic nanoparticles characterized by a carbon-enriched surface and a progressive increase in iron content toward the inner regions of the nanostructures. Interestingly, this type of nanostructure, characterized by functionalized carbonaceous species predominantly concentrated at the surface, is particularly promising for applications involving interactions with a wide variety of molecules, since these surface functional groups can enhance interfacial interactions, adsorption processes, and chemical compatibility. This behavior arises from the surface characteristics of carbon dots, which are typically rich in functional groups whose composition can be rationally tuned through both precursor selection and postsynthesis functionalization strategies. As a result, the interfacial properties of the material can be deliberately tailored to promote specific interactions with target molecules, making it especially attractive for applications such as adsorption, including pollutant removal.

Figure 2c–f present, respectively, the high-resolution XPS spectra of the C 1s, N 1s, O 1s, and Fe 2p signals. The high-resolution C 1s spectrum (Figure 2c) was deconvoluted into four components centered at 284.6, 285.7, 287.0, and 288.5 eV, assigned to C–C/C=C, C–N/C–OH/C–O–C, C=O/C=N (carbonyl or imine), and COOH (carboxylate/carboxylic) carbon species, respectively.^45,46^ The deconvoluted high-resolution N 1s spectrum shown in Figure 2d exhibited two components centered at 398.4 and 399.3 eV, corresponding to pyridinic/imine (C=N) and amine/amide (C–N) nitrogen species, respectively.^47^ The high-resolution O 1s spectrum (Figure 2e) was deconvoluted into four components located at 530.2, 531.1, 532.3, and 533.7 eV, assigned to Fe–O (lattice oxide), C=O/–COO (oxygen doubly bonded to carbon or carboxylate oxygen), Fe–OH/–OH (surface hydroxyl groups), and C–O/COOH (oxygen singly bonded to carbon in carboxylic or alcohol species), respectively.^46,48^ The Fe 2p spectrum was analyzed using a constrained multiplet-envelope approach based on established fitting procedures for iron oxides.^49,50^ In the fitted spectrum, the blue set of multiplet peaks corresponds to Fe_3_O_4_, whereas the brown set corresponds to γ-Fe_2_O_3_ (maghemite). In this model, the Fe 2p_3_/_2_ envelope is described as overlapping Fe^2+^ and Fe^3+^ multiplet contributions, rather than as isolated single components, reflecting the mixed-valence nature of magnetite and the characteristic multiplet splitting of iron oxides.^49,50^ The fitting procedure followed the semiempirical parameters reported for iron oxides and the recommendations for Fe 2p_3_/_2_ analysis in complex iron oxide systems. The best fit obtained for Fe_3_O_4_-MCDs indicated a predominantly magnetite-like mixed-valence surface, with fitted contributions of approximately 76.1% Fe_3_O_4_ and 23.9% γ-Fe_2_O_3_. These results suggest that the magnetic iron oxide fraction is mainly composed of Fe_3_O_4_, while the higher-binding-energy contribution is consistent with a secondary Fe­(III)-rich component, attributed to partial surface oxidation or maghemitization.

Taken together, the XPS findings are in good agreement with the FTIR spectrum, corroborating the presence of graphitic carbon domains, nitrogen-containing functionalities (C=N and C–N), oxygenated surface groups (C–O, O–H, and O–C=O), as well as Fe–O bonds characteristic of the Fe_3_O_4_ phase.

The zeta potential measurements of the Fe_3_O_4_-MCDs as a function of pH, shown in Figure S3 (Supporting Information), reveal a pronounced pH-dependent surface-charge behavior, with positive zeta potential values under acidic conditions and negative values under basic conditions. At acidic pH values, the positive zeta potential is attributed to the protonation of nitrogen-containing functionalities, such as amine and pyridinic groups identified in the N 1s spectrum, together with protonated surface hydroxyl groups mainly associated with the Fe_3_O_4_ phase in the nanohybrid. As the pH increases, the progressive deprotonation of carboxylic, hydroxyl, and Fe–OH surface groups leads to a continuous decrease in the zeta potential, which becomes negative above pH 7. This behavior is in good agreement with the oxygen-containing functionalities identified by FTIR and XPS, particularly carboxyl/carboxylate and hydroxyl groups.

Importantly, the observed pH-dependent zeta potential behavior may be particularly relevant for adsorption applications, as it enables adjustment of the solution pH to favor specific electrostatic interactions between the adsorbent and the adsorbate. In the case of an anionic dye such as Congo red, these interactions are expected to play an important role in the adsorption process.

### 3.2 Morphological, Structural, and Magnetic Properties

Figure S4 (Supporting Information) shows transmission electron microscopy (TEM) images of the Fe_3_O_4_-MCDs acquired at different lower magnifications. Interestingly, the sample consistently exhibited structures with similar morphology across all recorded images, indicating a relatively homogeneous distribution throughout the analyzed regions. As observed in Figure S4, the material appears arranged into grape-like assemblies composed of several smaller particles associated with one another. These assemblies may appear partially supported on the lacey carbon film of the grid (Figure S4a and b) or suspended over the ultrathin carbon film spanning the lacey carbon support (Figure S4c). These clusters may reflect an intrinsic tendency of the Fe_3_O_4_-MCDs to self-associate, possibly enhanced by drying-induced aggregation during TEM grid preparation. More importantly, throughout the entire sample, all of these assemblies are consistently composed of smaller, independent, and nearly spherical nanoparticles (Figure 3). Figure 3 presents the TEM images obtained at higher magnifications, together with the particle size distribution histogram and the selected area electron diffraction (SAED) pattern.

**Figure 3:**
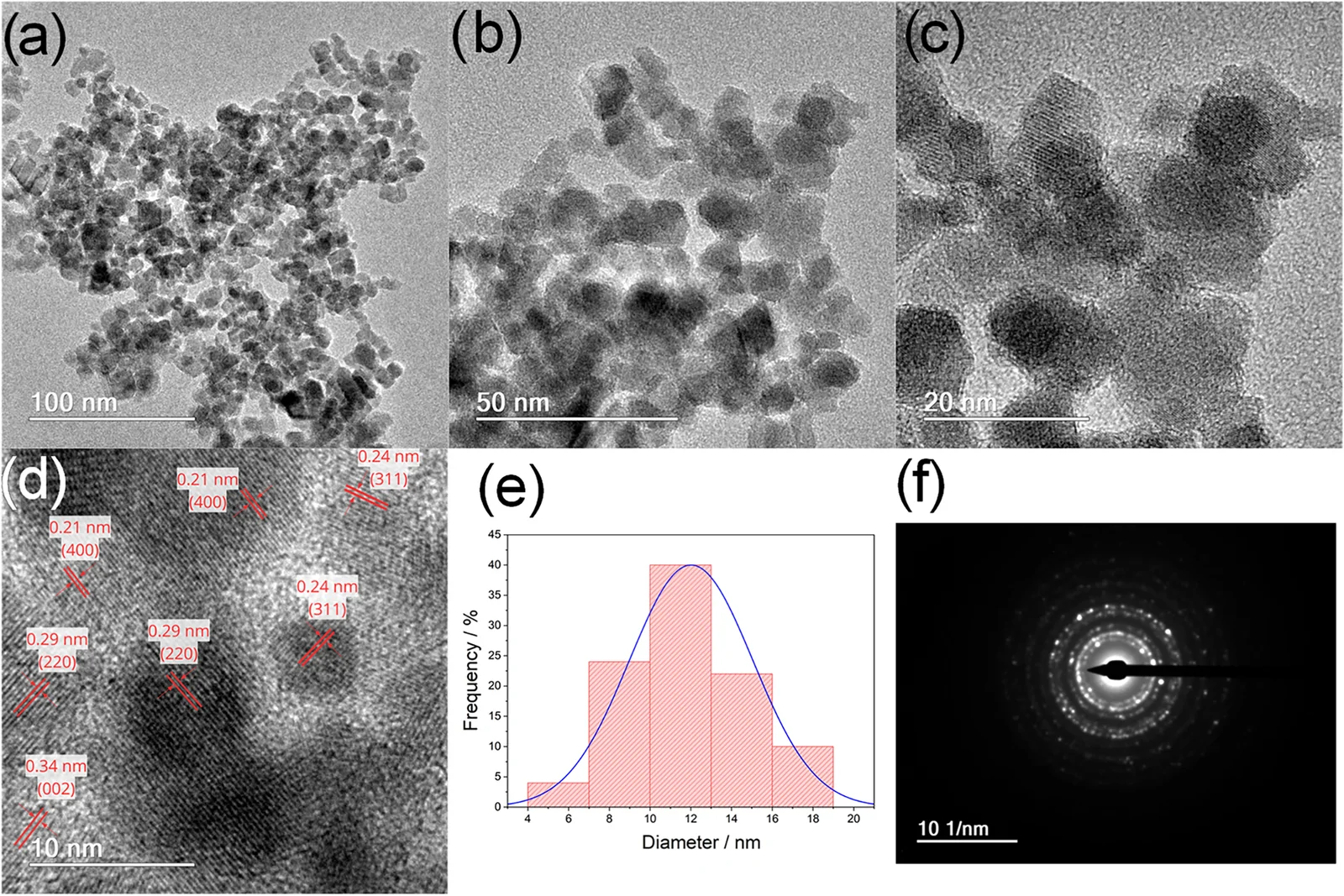
Transmission electron microscopy (TEM) images of the Fe_3_O_4_-MCDs at different magnifications (a-d). Image (d) reveals lattice fringes with interplanar spacings characteristic of graphitic domains and the magnetite phase. Panel (e) shows the particle size distribution histogram, while (f) presents the selected area electron diffraction (SAED) pattern.

The TEM images (Figure 3a–d) reveal nanoparticles with an approximately Gaussian size distribution, with an average diameter of 12 ± 3 nm, based on measurements of 100 particles (Figure 3e). It is worth noting that the size of the magnetic nanoparticles plays a crucial role in the development of superparamagnetic behavior, which is a key characteristic for the proposed adsorption and magnetic separation processes.

In addition, the TEM images suggest particles with a darker inner region and a lower-contrast outer layer (Figure 3c), consistent with a magnetite-rich core region and a carbon-enriched surface. This interpretation is supported by the XPS depth-profiling results, which showed a higher carbon contribution at the surface (before sputtering) and an increased iron signal after sputtering. HRTEM images (Figure 3c,d) further confirm the crystallinity of the particles, displaying interplanar spacings. In Figure 3d, the magnetite lattice planes of 0.29 nm (220), 0.24 nm (311), and 0.21 nm (400) are observed,^51^ along with the graphitic carbon interlayer spacing of 0.34 nm corresponding to the (002) plane.^52^ Additionally, the Selected Area Electron Diffraction (SAED) pattern (Figure 3f), acquired from a representative region of the Fe_3_O_4_-MCD nanohybrid sample, displays a ring-like diffraction profile, consistent with the polycrystalline nature of the magnetite phase. The observed ring positions can be indexed to the (311) and (400) crystallographic planes of Fe_3_O_4_. Superimposed discrete diffraction spots indicate the presence of locally well-crystallized domains within the nanoparticles.^53^ Moreover, a diffuse halo is visible in the central region of the pattern, which is attributed to the amorphous carbonaceous fraction, further supporting the coexistence of crystalline magnetite domains and a structurally disordered carbon-rich phase.^54,55^

Figure 4(a,b) shows the X-ray diffraction pattern and deconvoluted Raman spectrum results of the Fe_3_O_4_-MCDs.

**Figure 4:**
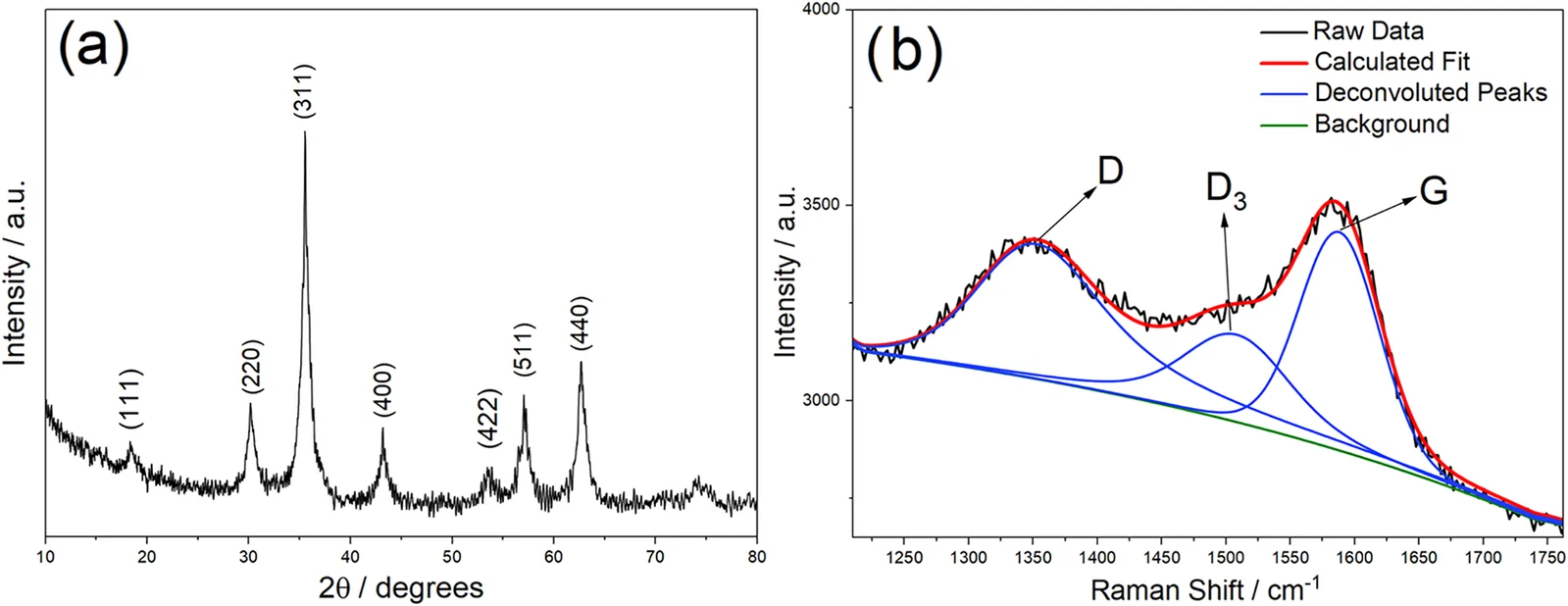
X-ray diffraction (XRD) pattern (a) and Raman spectrum (b) of Fe_3_O_4_-MCDs.

The XRD pattern of the Fe_3_O_4_-MCDs, shown in Figure 4a, exhibits diffraction peaks at 2θ values of 18.31°, 30.12°, 35.42°, 43.17°, 53.64°, 57.14°, and 62.62°, corresponding to the crystallographic planes (111), (220), (311), (400), (422), (511), and (440), respectively.^56^ The obtained diffraction profile can be assigned to the inverse cubic spinel structure of magnetite (Fe_3_O_4_), in agreement with JCPDS card No. 19–0629. The presence of well-defined and relatively narrow peaks suggests a good degree of crystallinity, efficient formation of Fe_3_O_4_ nanocrystals, and a low amorphous fraction in the inorganic phase. In addition, the noticeable broadening of the (311) peak is consistent with the nanometric nature of the crystallites. The average crystallite size was estimated to be 10.7 ± 0.6 nm using the Scherrer equation and averaging the values obtained for the (311), (220), (400), and (440) diffraction peaks.^57^ This value is in good agreement with the size distribution obtained from TEM images, centered at approximately 12 nm, further corroborating the nanometric character of the particles. The slightly smaller crystallite size estimated from XRD is also consistent with the fact that the Scherrer analysis primarily reflects the crystalline magnetite domains, whereas the TEM measurements include the overall dimensions of the Fe_3_O_4_-MCD nanohybrids, including the carbon-rich outer regions. Furthermore, a broad band together with a slight elevation of the baseline is observed in the low-angle region (2θ below 20°), which may be associated with the presence of a poorly ordered carbonaceous fraction.

To further verify the presence of the carbonaceous phase in the Fe_3_O_4_-MCD material and to better characterize this fraction, Raman spectroscopy was performed. The Raman spectrum of the Fe_3_O_4_-MCDs, shown in Figure 4b, confirms the presence of the carbonaceous component in the hybrid material, corroborating the coexistence of carbonaceous and magnetic phases in the sample. In addition, the obtained spectrum was deconvoluted and showed three components centered at 1348, 1502, and 1583 cm^–1^. The feature at 1348 cm^–1^ is assigned to the D band, which reflects disorder-induced vibrations associated with defective sp^2^ carbon domains.^58^ In contrast, the band at 1583 cm^–1^ corresponds to the G band (E_2_g stretching mode), which is characteristic of the in-plane stretching vibration of sp^2^-hybridized carbon atoms and indicates the presence of relatively more ordered graphitic domains.^59^ The intermediate component at 1502 cm^–1^ can be assigned to the D_3_ (or D″) band,^60,61^ commonly related to amorphous and highly disordered carbon species.^62^ Furthermore, the intensity ratio between the D and G bands (*I*_D_/*I*_G_) was determined to be 0.99, reflecting a carbon framework that combines defect-rich regions with locally preserved sp^2^-ordered domains, a structural feature commonly observed in carbon dot-derived materials.^59^ These results support the presence of a partially graphitized carbon phase, indicating the coexistence of conjugated sp^2^ domains with structural defects, surface functional groups, and amorphous carbon regions.

Figure 5a presents the magnetic hysteresis curve of the Fe_3_O_4_-MCDs recorded under an applied magnetic field ranging from −15 to +15 kOe, while Figure 5b shows an enlarged view of the central region. The magnetization curve displays a typical S-shaped profile and approaches saturation at relatively low field strengths, reaching a saturation magnetization (*M*_s_) of approximately 60 emu g^–1^, which confirms the strong magnetic response associated with the Fe_3_O_4_ phase. In addition, both the remanent magnetization (*M*_r_) and coercive field (*H*_c_) are nearly negligible, with values close to zero, as evidenced by the enlarged view in Figure 5b, indicating minimal magnetic hysteresis.^63^ Taken together, these results are characteristic of the superparamagnetic behavior of the material, in which the magnetization rapidly relaxes to nearly zero upon removal of the external magnetic field due to the fast randomization of magnetic moments.^64^

**Figure 5:**
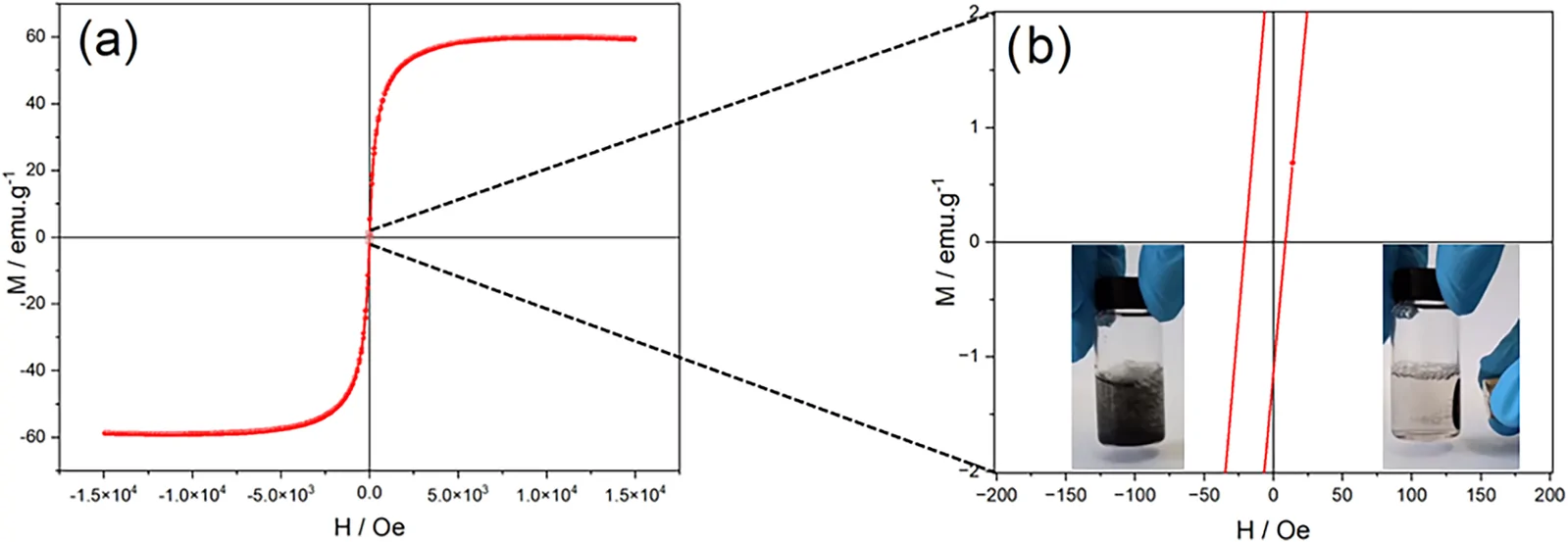
Magnetic hysteresis curve of the Fe_3_O_4_-MCDs (a) and enlarged view of the central region of the curve showing remanent magnetization (*M*_r_) and coercive field (*H*_c_) values close to zero (b). The insets in (b) show photographs of the Fe_3_O_4_-MCDs before (left) and after (right) the approximation of a magnet.

The inset photographs in the hysteresis plot further illustrate the magnetic responsiveness of the Fe_3_O_4_-MCDs sample before and after the application of an external magnetic field using a permanent magnet (see also Supporting Video 1). The rapid attraction of the material toward the magnet, together with its easy redispersion after removal of the field (near-zero values of *M*_r_ and *H*_c_), highlights its suitability for adsorption-based processes, particularly in environmental remediation applications, where fast magnetic separation and recovery of the adsorbent are highly desirable. The application of the obtained Fe_3_O_4_-MCD material in adsorption processes is discussed in the next section.

### 3.3 Adsorption Behavior and Performance Evaluation

In order to investigate the potential of MCDs as a versatile platform for environmental remediation applications, Congo red was selected as a model pollutant for the adsorption studies. The structural characteristics of these Fe_3_O_4_-MCDs, comprising a magnetite-rich interior and a carbon-rich outer region, enable tuning of the surface interaction properties through appropriate modification of the surface. The carbon-rich outer region can be tailored either by selecting suitable precursors for the carbonaceous phase or through postsynthesis functionalization strategies, allowing fine control over specific interactions of interest. As a result, these magnetic nanoparticles can exhibit remarkable versatility for environmental remediation, as their surface can be engineered to modulate affinity toward different pollutants and contaminants, thereby expanding their applicability across diverse environmental systems.

In addition, while adsorption in many remediation systems is strongly influenced by specific adsorbent–adsorbate interactions, the specific surface area also remains an important parameter. Therefore, its determination is relevant for the characterization of the material employed in the process. In this context, the N_2_ adsorption–desorption isotherm (Figure 6a) was used to investigate the textural properties of Fe_3_O_4_-MCD, providing relevant information about its adsorption behavior. The isotherm exhibits a typical type IV profile with an H3-type hysteresis loop, characteristic of mesoporous materials with textural porosity arising from the assembly of nanoparticles.^65^ The BET specific surface area was 107 m^2^ g^–1^. The total pore volume estimated by the Gurvich method was 0.431 cm^3^ g^–1^, while BJH analysis of the adsorption branch indicated a modal pore diameter of approximately 17 nm. The modal pore diameter corroborates the mesoporous nature of the material, while the relatively high pore volume suggests the presence of a significant amount of accessible void space within the structure. In addition, considering that the modal pore diameter (≈17 nm) is much larger than the estimated molecular length of Congo red (≈2.6 nm), steric hindrance is not expected to significantly affect dye molecular diffusion within the mesoporous structure. Therefore, the mesoporous network and interparticle voids generated by the noncompact assembly of nanoparticles can readily accommodate CR molecules and facilitate their diffusion toward the adsorption sites.

**Figure 6:**
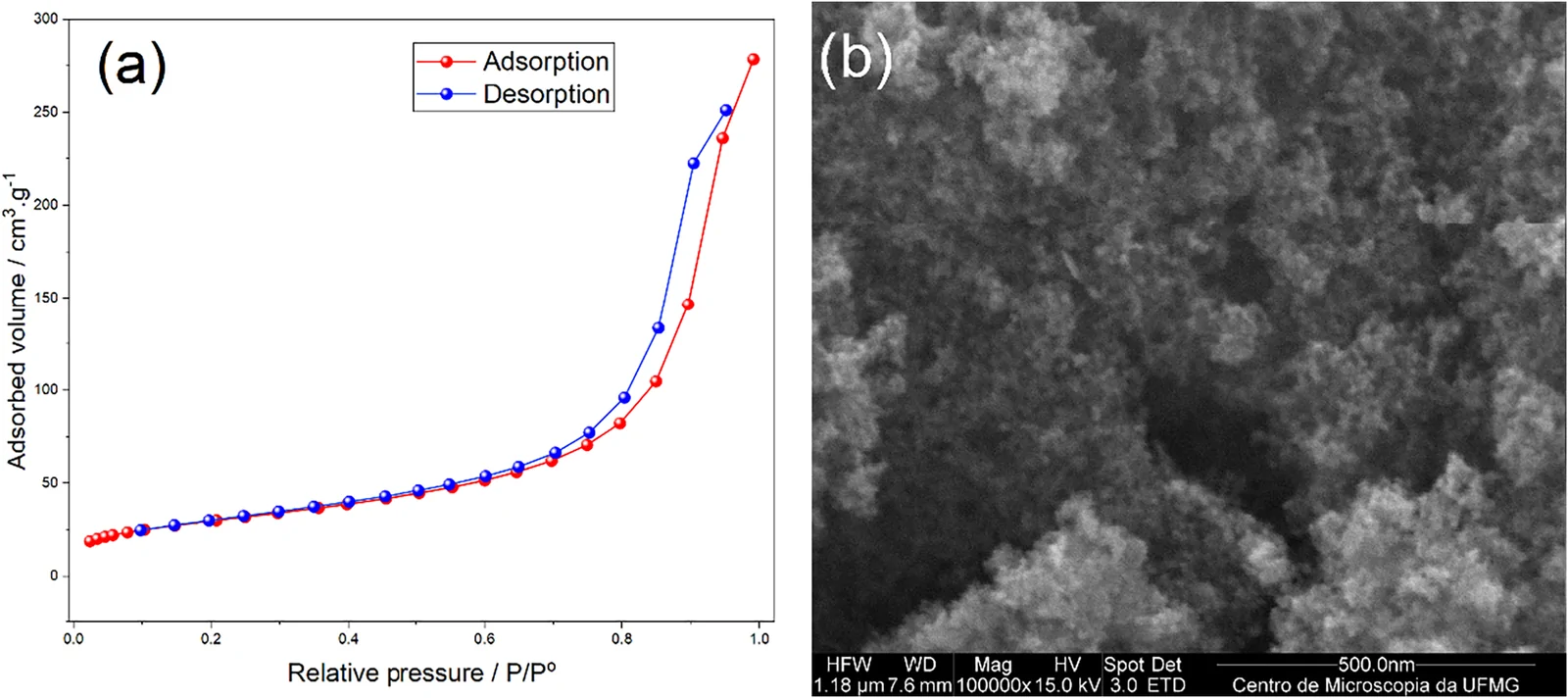
Textural and morphological characteristics of Fe_3_O_4_-MCD. N_2_ adsorption–desorption isotherm (a) and SEM image of Fe_3_O_4_-MCD (b).

To gain further insight into the origin of the mesoporous structure identified by N_2_ adsorption measurements, scanning electron microscopy (SEM) images were acquired to investigate the morphological organization of the Fe_3_O_4_-MCD material. The SEM image (Figure 6b) shows a rough and heterogeneous surface of the Fe_3_O_4_-MCD, with a noncompact packing of primary nanoparticles forming a cauliflower-like aggregated morphology and displaying interstices between the aggregates. The loose packing of the Fe_3_O_4_-MCD nanoparticle assemblies generates interparticle voids that may contribute to the accessible surface area measured by N_2_ adsorption and can play an important role in the adsorption of contaminants. shows an additional SEM image of the same sample at lower magnification. In addition, the cauliflower-like morphology observed by SEM is consistent with the primary quasi-spherical nanoparticles and the grape-like assemblies previously identified in the TEM images (Figures 3a–c and S4, respectively), indicating that the larger structures are formed by the assembly of smaller primary particles and revealing a hierarchical organization of Fe_3_O_4_-MCD across different length scales. Overall, the morphological features observed by TEM and SEM are highly consistent with the type IV isotherm profile and the H3-type hysteresis loop, indicating that the mesoporosity of Fe_3_O_4_-MCD arises from the noncompact assembly of nanoparticles.

Furthermore, as electrostatic interactions are among the main driving forces in adsorption processes, this study aimed to enhance the interaction between the pollutant and the MCDs mainly through electrostatic attraction. The Fe_3_O_4_-MCDs exhibit a positive zeta potential at low pH values (Figure S3), whereas Congo red, a sulfonated azo dye, remains negatively charged over a broad pH range due to the dissociation of its sulfonic acid groups.^66^ Thus, adsorption studies were carried out at various pH values, and the results are presented in Figure S6. The enhanced adsorption at pH 3 can be attributed to electrostatic interactions between the highly positively charged Fe_3_O_4_-MCD surface and the negatively charged sulfonate groups of CR. Congo red contains two sulfonate groups (−SO_3_^-^), which remain ionized over a broad pH range, thereby maintaining an overall negative charge even under acidic conditions, despite possible partial protonation of its amino groups. The improved adsorption observed at pH 3 is consistent with previous reports on carbon-based adsorbents under acidic conditions for CR.^66^ In addition, this behavior highlights the pivotal contribution of electrostatic attraction to the adsorption of CR onto Fe_3_O_4_-MCD. Nevertheless, additional interactions may also contribute to the adsorption mechanism, including hydrogen bonding and π-π stacking interactions. These aspects will be further discussed after the quantitative adsorption studies.

Figure 7a shows the percentage removal of CR as a function of time at different initial dye concentrations. The removal percentage values were calculated according to eq ([Disp-formula eq2]), and the curves reveal rapid adsorption within the first 30 min, followed by a gradual approach to equilibrium. Removal percentages of approximately 94% and 98% were achieved at equilibrium for initial concentrations of 5.65 mg L^–1^ and 15.03 mg L^–1^, respectively.

**Figure 7:**
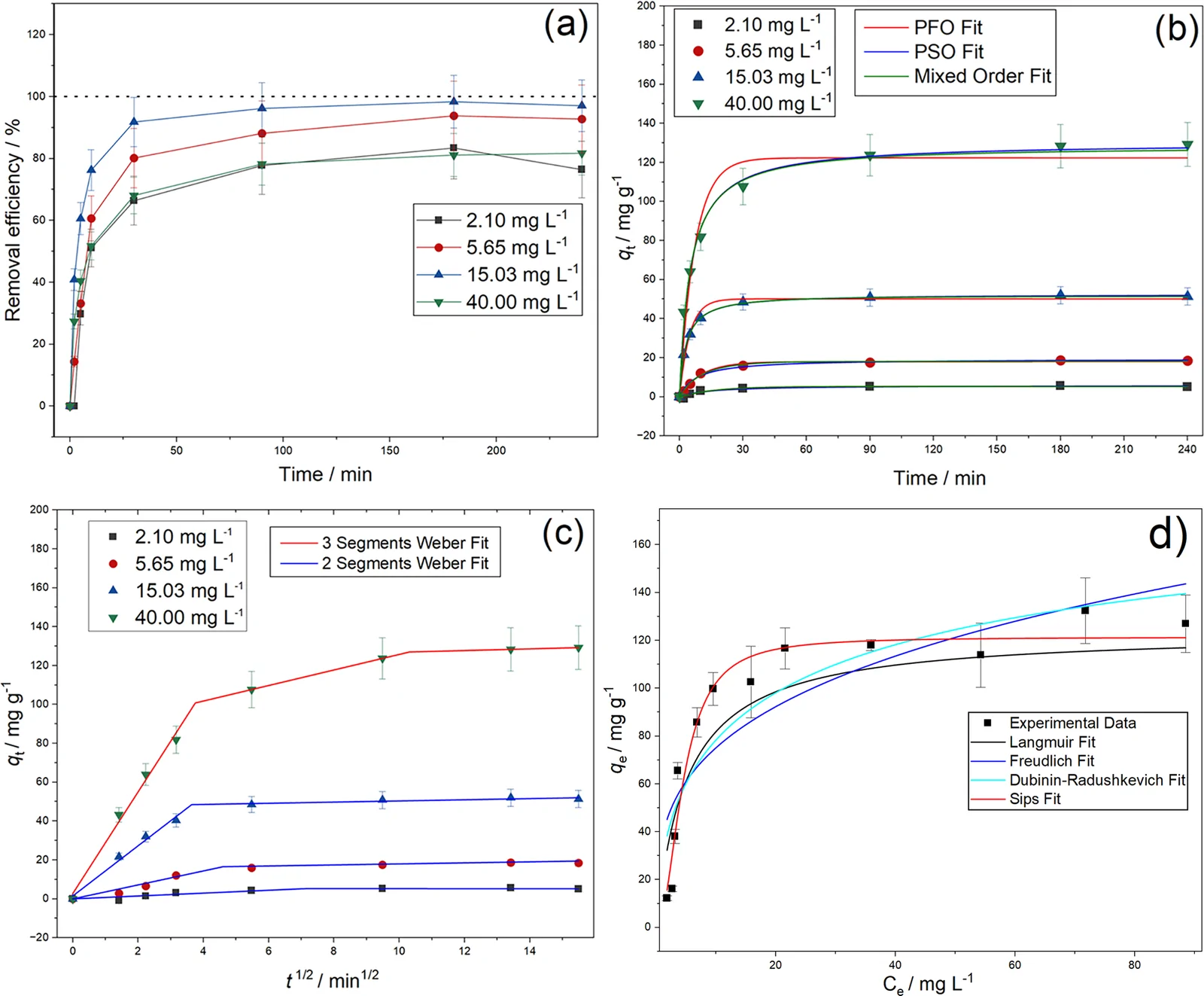
Removal efficiency (%) as a function of time at different initial CR concentrations (a), adsorption capacity (*q_t_*) versus time with different kinetic model fits (b), adsorption capacity (*q_t_*) as a function of square root of time (*t* ^1/2^) fitted with intraparticle diffusion model (c), and adsorption isotherms fitted with the Langmuir, Freundlich, Sips, and D-R models (d). Error bars represent standard deviations from triplicate measurements.

Supporting Video 2, presented in a time-lapse format, illustrates the visual progression of the adsorption of a CR solution with an initial concentration of 15 mg L^–1^ by Fe_3_O_4_-MCD. The solution becomes almost completely decolorized after approximately 45 min, in agreement with the results shown in Figure 7a.

Figure 7a also shows that a lower removal efficiency is observed at lower initial dye concentrations (2.10 mg L^–1^). This behavior can be attributed to the reduced driving force for mass transfer under these conditions, which limits the interaction between dye molecules and the available active sites on the surface of the MCDs. As a result, fewer adsorption events occur over time, leading to lower removal percentages under these dilute dye concentrations. On the other hand, at the highest initial dye concentration (40.00 mg L^–1^), a decrease in removal percentage is also observed, which can be attributed to the limited adsorption capacity of the system, as the increased number of dye molecules enhances competition for the available active sites on the MCD surface.

Figure 7b presents the adsorption capacity (*q_t_*) as a function of time at different initial CR concentrations, calculated using eq ([Disp-formula eq3]), together with kinetic fits based on the pseudo-first order (PFO), pseudo-second order (PSO), and mixed 1,2-order models. Figure 7c presents the corresponding plots of adsorption capacity (*q_t_*) versus the square root of time (*t* ^1/2^), fitted according to the intraparticle diffusion model.

First, a rapid increase in *q_t_* is observed within the first minutes of contact, followed by a gradual approach to equilibrium, similar to the behavior observed for the removal percentage. In addition, the adsorption capacity increases with initial dye concentration, reaching higher values at the highest concentrations evaluated. This behavior can be attributed to the increased mass transfer driving force, which enhances the diffusion of dye molecules toward the available active sites on the Fe_3_O_4_-MCD surface. After approximately 90 min, the curves reach a plateau, indicating the establishment of adsorption equilibrium associated with the progressive occupation of the active sites. Furthermore, the *q_t_* value for the 40.00 mg L^–1^ dye concentration reaches approximately 124 mg g^–1^ at around 90 min of contact time.

In general, different kinetic models can be used to describe the adsorption kinetics and assess the adsorption rate. A comprehensive summary of the fitting results, including *R* ^2^ and χ_red_^2^ values and the corresponding kinetic model parameters (*q*_e_, *k*_1_, *k*_2_, *f*_1_, *f*_2_) for each model as a function of the initial concentration, is presented in Table S1. Among the kinetic models evaluated using nonlinear fitting, the pseudo-first order (PFO) model provided the best fit for the initial dye concentrations of 2.10 and 5.65 mg L^–1^, whereas the pseudo-second order (PSO) model best described the adsorption kinetics at 15.03 and 40.00 mg L^–1^, as indicated by the higher *R* ^2^ and lower χ_red_^2^ values (Table S1).

Notably, the 1,2-MO model estimated an equilibrium adsorption capacity (*q*_e_) of 127.1 ± 9.1 mg g^–1^ at an initial solute concentration of 40.00 mg L^–1^, a value very similar to the experimental equilibrium adsorption capacity (*q*_e,exp_ = 128.8 ± 0.6 mg g^–1^). It is important to note that the mixed 1,2-order model is considered here as a phenomenological tool to capture the complex kinetic behavior and multiple kinetic regimes observed in the system, rather than as a fundamental mechanistic rate law or direct evidence of a specific adsorption mechanism. In the 1,2-MO model, the parameters *f*_1_ and *f*_2_ represent the relative contributions of the first- and second-order terms, respectively. The obtained values indicate that the kinetic behavior was predominantly described by the first-order term at lower CR concentrations, whereas the contribution of the second-order term became more pronounced at higher concentrations.

Although Fe_3_O_4_-MCD does not possess a well-defined porous structure like activated carbons, as its porosity mainly arises from the assembly of nanoparticles, the intraparticle diffusion model was applied to analyze the first stage of CR adsorption onto Fe_3_O_4_-MCD at different initial dye concentrations (Figure 7c and Table S1). Successive 2.7-fold increases in the initial CR concentration, from 2.10 to 40.00 mg L^–1^, progressively increased the intraparticle diffusion rate constant (*k*_id,1_). However, the incremental increase in *k*_id,1_ became progressively smaller (4.8-, 3.6-, and 2.0-fold), indicating that the influence of the initial dye concentration on the initial diffusion rate became less pronounced at higher concentrations. This indicates that the transport of CR molecules from the bulk solution to the Fe_3_O_4_-MCD nanoparticle aggregates becomes progressively faster as the initial CR concentration increases. However, because the increase in *k*_id,1_ becomes progressively less pronounced with increasing initial CR concentration, this behavior likely reflects saturation and transport limitations. These effects may arise from (i) competition among CR molecules for active sites and the progressive occupation of the surface and pores, and (ii) the nonporous structure formed by assembled nanoparticles, suggesting that intraparticle diffusion is not the sole rate-controlling step. Contributions from external film diffusion, surface heterogeneity, and possible particle agglomeration at higher CR concentrations are also plausible. Therefore, the results suggest that CR adsorption onto Fe_3_O_4_-MCD involves multiple kinetic regimes.

Figure 7d shows the experimental adsorption isotherm along with the nonlinear fits of different isotherm models, describing the relationship between the equilibrium adsorption capacity (*q*_e_) and the equilibrium dye concentration in solution (*C*_e_). All parameters derived from the fitting of the different models are summarized in Table S2. Among the models evaluated, the Sips model showed the best performance, with an *R* ^2^ value of 0.9545, indicating better agreement with the experimental data. This model involves a greater number of adjustable parameters, as it is a three-parameter equation, whereas the Langmuir and Freundlich models contain only two adjustable parameters. In addition, the Sips isotherm is an empirical model that combines the characteristics of the Langmuir and Freundlich isotherms. The statistical agreement between the Sips model and the experimental data is consistent with adsorption on a heterogeneous surface, while its asymptotic behavior accounts for saturation at high concentrations, avoiding the unrealistic unlimited increase predicted by the Freundlich model. Fe_3_O_4_-MCD nanoparticles exhibit a heterogeneous surface (Figure 6) with different adsorption sites, including nitrogen-containing functionalities (pyridinic C=N and amine C–N), oxygen-containing functionalities (aliphatic and phenolic C–OH, carboxylic O–C=O, and Fe–OH groups), and aromatic structures. The Sips model has been successfully applied to describe adsorption on heterogeneous surfaces, which is consistent with the structural complexity of Fe_3_O_4_-MCD nanoparticles and the presence of multiple surface functionalities. Although the Dubinin–Radushkevich model did not provide the best fit to the equilibrium data, its characteristic energy parameter (*E*_D–R_) was also evaluated. Using *C*_s_ = 1000 mg L^–1^, corresponding to the CR saturation concentration reported by the dye supplier, the *E*_D–R_ value was 8.94 ± 0.81 kJ mol^–1^. This value falls within the range commonly associated with physical adsorption (8–16 kJ mol^–1^). However, it is important to mention that the *E*_D‑R_ value obtained here should be interpreted only as a characteristic parameter of the D-R fit, consistent with the adsorption energy estimated by the model, rather than as conclusive evidence of a specific adsorption mechanism.

Additionally, the initial slope of the adsorption isotherm and the Sips model parameter (*b* = 0.23 ± 0.03 L mg^–1^), Figure 7d, are consistent with the favorable adsorption behavior observed at low equilibrium concentrations, which is also reflected in the fast adsorption kinetics (Figure 7b). Furthermore, the Sips (*Q*_max_ = 121.3 ± 5.0 mg g^–1^) model provided excellent agreement with the experimental maximum adsorption capacity (*Q*_max,exp_ = 121.6 ± 7.8 mg g^–1^).

In addition, since the highest adsorption was observed at pH 3, we also investigated the possibility of partial iron leaching from the Fe_3_O_4_-MCD nanohybrid using flame atomic absorption spectrometry (FAAS). Four experimental conditions were evaluated. In the first, 8 mg of Fe_3_O_4_-MCD were digested with concentrated HNO_3_/HCl (3:1, v/v) at 40 °C for ∼30 min, and the resulting Fe concentration was determined by FAAS as the total Fe content of the nanohybrid. In the second condition, 8 mg of Fe_3_O_4_-MCD were agitated in aqueous media at pH 3 (HCl) for up to 240 min, under the same conditions used in the adsorption experiments. In the third condition, the same amount of nanohybrid was agitated for 240 min at pH 8 (NaOH). An analytical control was also prepared with deionized water at pH 3, without the nanohybrid, to assess background Fe in the water.

In the first experiment, the acid-digested Fe_3_O_4_-MCD solution was analyzed by FAAS to determine the total Fe content. In the second and third experiments, after agitation, the Fe_3_O_4_-MCD was magnetically separated, and the supernatants were analyzed by FAAS to determine the Fe concentration in the aqueous phase. Additionally, at pH 3, contact times of 30 and 120 min were evaluated alongside 240 min to assess Fe leaching over time. All conditions were tested in triplicate (*n* = 3).

The results showed that Fe leaching after 240 min was 1.10 ± 0.31% at pH 8 and 4.17 ± 0.20% at pH 3, corresponding to 95.83 ± 0.20% of Fe remaining associated with the Fe_3_O_4_-MCD nanohybrid under the acidic conditions (pH 3). The results of Fe leaching over time (at pH 3) showed an increase from 2.53 ± 0.17% at 30 min to 3.69 ± 0.31% at 120 min. It is important to note that these results are particularly relevant, as the adsorption system reached equilibrium within 120 min, when Fe leaching was still lower than that observed after 240 min. The results obtained here are in good agreement with some reports in the literature on Fe leaching from nanomagnetite and magnetite-based nanohybrids under acidic conditions.^67,68^ Overall, the FAAS results demonstrated limited Fe leaching under the experimental conditions, including those used for Congo red adsorption at pH 3.

Figure S7 shows adsorption and readsorption capacities over successive regeneration cycles calculated using eqs S1–S7. To promote CR desorption, the pH of the Fe_3_O_4_-MCD suspensions was increased using either KOH or NaOH (0.01 mol L^–1^), followed by washing with ultrapure water. Overall, the readsorption experiments yielded slightly higher adsorption capacities when NaOH was used as the desorbing agent. The most pronounced decrease in adsorption capacity occurred after the first regeneration cycle, whereas the adsorption performance remained comparatively more stable throughout the subsequent cycles. Although a gradual decline in adsorption capacity was observed, particularly after the first regeneration cycle, the Fe_3_O_4_-MCD nanohybrid retained a significant fraction of its adsorption performance, demonstrating its potential for repeated use. The pronounced loss observed after the first reuse cycle suggests that a fraction of the adsorption sites is not completely regenerated during the desorption process, whereas the remaining sites exhibit reasonable stability throughout the subsequent cycles. In other words, the smaller variation observed during the subsequent cycles suggests that a significant fraction of the active adsorption sites remains accessible and functional throughout the reuse process. Finally, the decrease in adsorption capacity can be related to the interactions established between CR molecules and the adsorption sites on Fe_3_O_4_-MCD, which may hinder the complete regeneration of some adsorption sites during the desorption process. This may have hindered complete desorption of CR molecules from Fe_3_O_4_-MCD under the mild regeneration conditions employed. In addition to the possible blocking of active sites due to these interactions, mild alteration or degradation of surface functional groups during the desorption treatment cannot be excluded and was further investigated by FTIR, as described later in this section. The interactions between CR molecules and adsorption sites on Fe_3_O_4_-MCD are mainly driven by electrostatic attraction, as also indicated by the adsorption behavior observed at different pH values, although additional contributions from hydrogen bonding and other interactions may also be involved.

To further elucidate the interactions between Fe_3_O_4_-MCD and Congo red, the FTIR spectra of neat Fe_3_O_4_-MCD and Congo red were compared with those of Fe_3_O_4_-MCD after adsorption of the dye and subsequent desorption (Figure 8).

**Figure 8:**
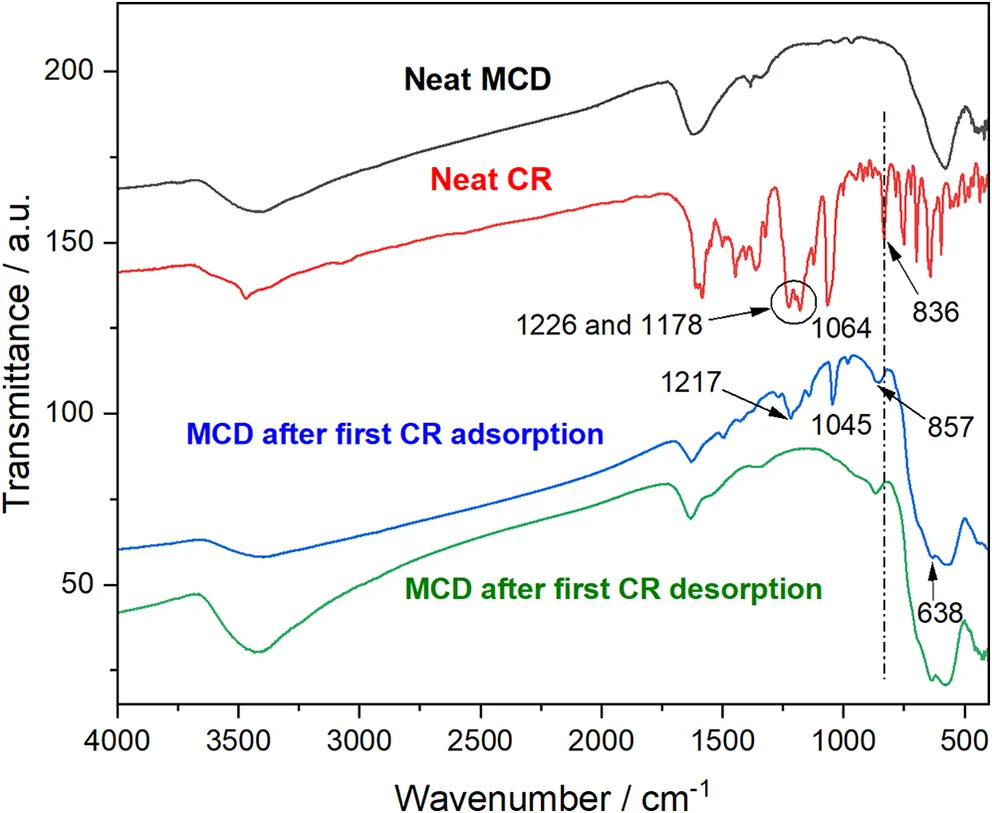
FTIR spectra of pristine Fe_3_O_4_-MCD, pristine CR, Fe_3_O_4_-MCD after CR adsorption, and Fe_3_O_4_-MCD after dye desorption.

The FTIR spectrum of neat Congo red exhibits characteristic bands at 1585 cm^–1^, attributed to azo (−N=N−) and aromatic C=C stretching vibrations, 1350 cm^–1^ assigned to C–N stretching of aromatic amine groups, and a broad band in the 3467–3475 cm^–1^ region associated with N–H stretching vibrations of primary amines.^69,70^ In addition to these bands, characteristic signals at 1226 and 1178 cm^–1^, assigned to the asymmetric stretching vibrations of sulfonate groups (SO_3_^–^),^71^ as well as the band at 1064 cm^–1^ corresponding to the symmetric stretching of the same group,^69,71^ and the band at 836 cm^–1^ attributed to aromatic C–H out-of-plane bending vibrations,^69^ are observed in the FTIR spectrum of neat Congo red (Figure 8). After the first adsorption of CR on the MCD surface, the characteristic bands associated with the asymmetric stretching vibrations of sulfonate groups exhibit significant changes, merging from two bands into a single band at 1217 cm^–1^, while the band corresponding to the symmetric stretching vibration of SO_3_^–^ shifts from 1064 to 1045 cm^–1^. In addition, the band assigned to the out-of-plane bending vibration of aromatic C–H bonds shifts from 836 to 857 cm^–1^. The corresponding band shifts and spectral changes are highlighted in Figure 8. The shifts and spectral changes observed for the sulfonate vibrations indicate a significant perturbation of these groups upon adsorption, evidencing their direct involvement in the adsorption, likely through electrostatic interactions and hydrogen bonding with functional groups on Fe_3_O_4_-MCD. Furthermore, the shift of the aromatic vibration from 836 to 857 cm^–1^ indicates changes in the electronic environment of the aromatic rings, suggesting the contribution of π–π interactions between the conjugated structure of CR molecules and the carbonaceous domains of the adsorbent. As expected, these findings indicate that multiple interactions are involved in the adsorption of Congo red on Fe_3_O_4_-MCD. After desorption, most of the characteristic FTIR bands of CR disappeared. However, weak residual bands at approximately 857 and 638 cm^–1^, which coincide with bands observed for neat Congo red, remained detectable, suggesting the presence of a small amount of residual dye on the Fe_3_O_4_-MCD surface.

To further investigate and understand the adsorption–desorption processes, a second adsorption–desorption cycle was performed, using 0.01 mol L^–1^ NaOH as the desorbing solution, and the results are presented in Figure S8. In the second adsorption process, the same main bands observed after the first process, at 1217, 1045, and 857 cm^–1^, are again observed. After the second desorption process, the results are similar to those observed after the first one, with the persistence of the bands at 857 and 638 cm^–1^, attributed to the dye. These results corroborate those obtained from the reuse experiments, since the presence of residual dye on the Fe_3_O_4_-MCD surface is already observed after the first desorption. After the second desorption, the persistence of these bands indicates that a small amount of dye also remains associated with the surface, suggesting that the occupation of some adsorption sites by CR molecules that are not completely desorbed may contribute to the greater loss in adsorption capacity observed after the first cycle. In addition, the possibility of mild alteration or degradation of functional groups present on the MCD surface cannot be ruled out, since some bands associated with these groups, particularly in the 1400–950 cm^–1^ region (Figure S9), show decreased intensity or disappear after the adsorption–desorption processes. Therefore, the greater loss in adsorption capacity observed after the first cycle may be related to the combined contribution of the occupation of some adsorption sites by residual CR molecules and possible alterations in surface functional groups involved in the CR-MCD interactions, with these effects being more pronounced during the first regeneration cycle. In subsequent cycles, the smaller variation in adsorption capacity may be associated with the fact that both the fraction of sites occupied by residual dye and the possible alterations in the functional groups tend to be more limited.

Based on the adsorption and FTIR results, the proposed interactions involved in the adsorption mechanism are schematically illustrated in Figure 9. The adsorption process is likely governed by physisorption involving electrostatic interactions between the protonated amine groups from pyridinic and amine moieties, as well as hydroxyl groups coordinated to Fe atoms, and the charged functional groups of Congo red. In addition, hydrogen bonding interactions may occur between aliphatic and phenolic hydroxyl, carboxyl, and/or amine groups on the Fe_3_O_4_-MCD surface and the functional groups of Congo red, together with π–π stacking interactions between the conjugated sp^2^ carbon domains of the MCD surface and the aromatic rings of the dye.

**Figure 9:**
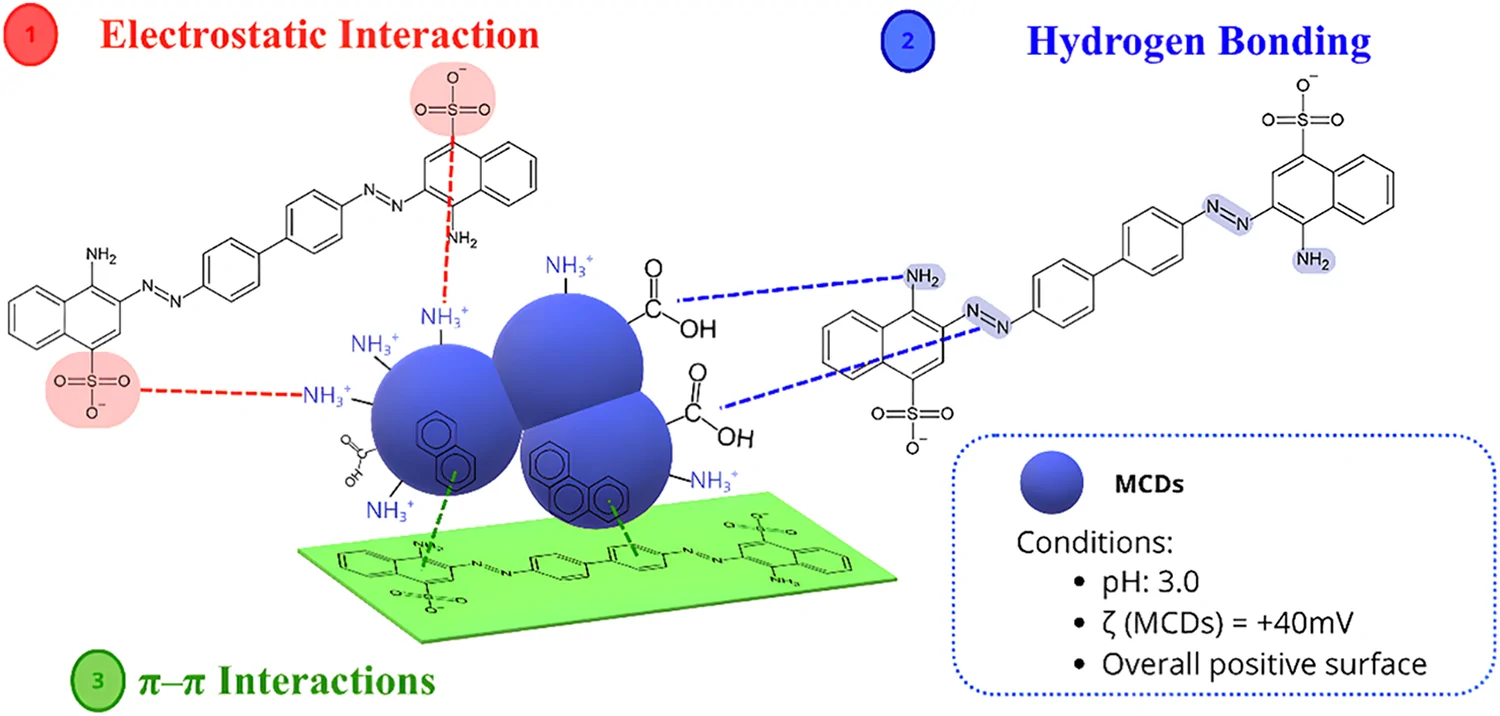
Schematic representation of the proposed interaction mechanisms between adsorption sites on Fe_3_O_4_-MCD particles and Congo red molecules, highlighting electrostatic interactions (red), hydrogen bonding (blue), and π–π interactions (green).

The presence of different surface functionalities on Fe_3_O_4_-MCD may contribute to the observed adsorption behavior. The superior fit of the Sips model is consistent with heterogeneous adsorption behavior, while the D–R model also provided a suitable empirical description of the equilibrium data. These results are consistent with the structural complexity of Fe_3_O_4_-MCD and the coexistence of different surface functionalities capable of interacting with Congo red.

Table 1 compiles different magnetic nanomaterials reported in the literature for CR removal, allowing a comparison among systems with distinct architectures, compositions, and surface functionalities. The values reported in the table correspond to experimentally determined data. Overall, a wide variation in adsorption capacities is observed, indicating that the adsorption performance is primarily governed by the surface structure and the density of available active sites.

**Table 1:** Comparison of Adsorption Capacity, Saturation Magnetization, Particle Size, and Adsorption Mechanisms of Magnetic Nanomaterials Reported in the Literature for Congo Red Removal

Adsorbent name	Adsorption capacity/mg g^–1^	Saturation magnetization/emu g^–1^	Average particle size/nm	Main adsorption mechanism	Reference number
N-CDs/CuFe_2_O_4_	15.85	44.98	200 nm	Electrostatic + H-Bonding + π–π	^72^
Fe_3_O_4_@C@C	19.0	72.3	200–300 nm	Chemisorption	^73^
MK composite	45.0	94.43	17.36 nm by Scherrer	Physisorption + Intraparticle Diffusion	^74^
Fe_3_O_4_/PVP	60.91	not reported	35 nm by Scherrer	Electrostatic Interaction + Heterogeneous Adsorption	^75^
CaFe_2_O_4_ NPs	105.34	1.83	37 nm Scherrer; 100–200 nm TEM	Electrostatic Attraction + H-Bonding	^76^
CoFe_2_O_4_@HaP	15.25	34.83	57.88 nm (Scherrer)	Chemisorption	^77^
CoFe_2_O_4_	136.13	33.6	∼3 nm TEM; ∼4 nm Scherrer	Electrostatic Interaction	^78^
FTT	74.01	39	47 nm	Electrostatic Interaction + π–π Interaction	^79^
Fe_3_O_4_@BTCA nanoparticles	192	not reported	15–20 nm	H-bonding + electrostatic + π–π	^80^
Fe_3_O_4_/NiO nanocomposite	172.3	24.75	50 nm (SEM)	Electrostatic + surface adsorption	^81^
Fe_3_O_4_-APTES-EDTA	∼ 82.0^a^	61.5	∼30 nm by Scherrer	Multilayer Adsorption	^82^
Fe_3_O_4_/chitosan	∼35.5^a^	not reported	not reported	Chemisorption + diffusion	^83^
Fe_3_O_4_-MCD	121.6	60	12 nm	Electrostatic + H-bonding + π–π	This work

a Values were estimated from the experimental data points shown in the corresponding plots, because the exact numerical values were not explicitly tabulated or reported in the text.

The adsorption capacity of 121.6 mg g^–1^ achieved by the Fe_3_O_4_-MCD synthesized in this work is within the upper range of the selected literature examples, although direct comparison is limited by differences in experimental conditions. Importantly, this capacity is achieved while maintaining a high saturation magnetization of 60 emu g^–1^. Although some materials exhibit higher adsorption capacities, such as Fe_3_O_4_@BTCA (192 mg g^–1^), these systems typically rely on highly specific surface functionalization (in this case, with benzenetetracarboxylic acid), requiring considerably more complex synthesis routes. Other materials, such as CoFe_2_O_4_ and Fe_3_O_4_/NiO, exhibit saturation magnetization values significantly lower than that of Fe_3_O_4_-MCD, a critical parameter for ensuring rapid and efficient magnetic separation in practical applications. Furthermore, the average particle size of 12 nm, among the smallest reported in the literature, may enhance the accessibility of adsorption sites through the formation of interparticle voids between the nanoparticles. In addition, the combination of three adsorption mechanisms in Fe_3_O_4_-MCD, namely electrostatic interactions, hydrogen bonding, and π–π interactions, enables multisite interactions between the material and CR molecules.

Overall, these results position Fe_3_O_4_-MCD as an efficient, magnetically recoverable adsorbent with a straightforward synthesis route for the removal of anionic dyes from aqueous matrices. More specifically, within the class of magnetic carbon dots, the properties achieved by Fe_3_O_4_-MCD may pave the way for the application of colloidal magnetic carbon dot nanoparticles in environmental remediation.

## 4 Conclusion

A straightforward synthetic strategy employing only AFC and EDA as precursors was successfully used to integrate nanocarbon and magnetite phases into Fe_3_O_4_-MCDs. The synthesized nanomaterial exhibited the characteristic optical properties of carbon dots, with a surface enriched with hydroxyl, carboxyl/carboxylate, and nitrogen-containing functionalities, which may play an important role in promoting interfacial interactions between Fe_3_O_4_-MCDs and different substances. These nanoparticles exhibited a homogeneous size distribution, with an average diameter of approximately 12 nm, and, at a larger scale, a hierarchical grape-like nanoarchitecture formed by nanoparticle assemblies. These assemblies generated interparticle voids, contributing to the high specific surface area of 107 m^2^ g^–1^ and a total pore volume of 0.431 cm^3^ g^–1^. In addition, the individual nanoparticles exhibited a nanoarchitecture consisting of an Fe_3_O_4_-rich inner region surrounded by a functionalized carbon-rich outer layer. They also exhibited a high saturation magnetization (∼60 emu g^–1^) together with negligible remanence and coercivity, consistent with superparamagnetic-like behavior at room temperature.

As a proof-of-concept for environmental remediation, Fe_3_O_4_-MCDs demonstrated efficient CR removal, reaching an adsorption capacity of 121.6 mg g^–1^. Kinetic analysis using the mixed 1,2-order model revealed the transition from predominant pseudo-first order to pseudo-second order behavior with increasing CR concentration, while intraparticle diffusion analysis indicated that diffusion contributes to the adsorption process but does not solely govern the adsorption kinetics, suggesting the involvement of multiple steps in the adsorption process. Equilibrium studies indicated that the Sips model provided the best fit to the experimental data, which is consistent with the heterogeneous nature of the adsorption surface. Furthermore, FTIR analyses before and after adsorption indicated that pollutant uptake results from multiple interactions involving electrostatic attraction, hydrogen bonding, and π–π interactions.

Finally, compared with previously reported magnetic carbon-based materials, the Fe_3_O_4_-MCD nanohybrid combines facile synthesis, high saturation magnetization, superparamagnetic behavior, and efficient pollutant removal, representing a multifunctional magnetically recoverable platform for environmental remediation applications. More broadly, the tunable surface chemistry of MCDs enables selective interactions with different compounds and pollutants, suggesting that their applicability may extend beyond dye removal, thereby paving the way for the use of MCDs in a broader range of environmental remediation strategies.

## Supplementary Material







## Notes

Magnetic responsiveness of Fe_3_O_4_-MCDs to a permanent magnet (Video S1) (MP4)
Time-lapse showing CR solution adsorption by Fe_3_O_4_-MCDs (Video S2) (MP4)
Calculation of desorption efficiency and reusability; fluorescence quantum yield determination (Figure S1); XPS depth profiling (Figure S2); zeta potential as a function of pH (Figure S3); low-magnification TEM and SEM images (Figures S4 and S5); effect of pH on Congo red adsorption (Figure S6); kinetic and adsorption isotherm parameters (Tables S1 and S2); adsorption capacity over successive regeneration cycles (Figure S7); FTIR spectra of Fe_3_O_4_-MCDs before and after adsorption and desorption cycles (Figures S8 and S9) (PDF)
