# Supplementary material for: Straightforward Synthesis of Superparamagnetic Fe3O4‑Carbon Dot Nanohybrids for Environmental Remediation: A Model Study Using Congo Red
Source: ACS Omega. 2026 Oct 28;11(44):68946–62. doi: 10.1021/acsomega.6c08673 (PMC13756651; doi:10.1021/acsomega.6c08673)
Supplement: Supplementary file 1 [file ao6c08673_si_003.pdf]

Straightforward synthesis of superparamagnetic Fe_3O_4-carbon dot nanohybrids for environmental remediation: a model study using Congo red

Arthur Hegermann Ferreira [a], Pedro Lana Gastelois [b], Leandro Vinícius Alves Gurgel [c], Fabiano Vargas Pereira [a,]*

[a] *Department of Chemistry - Universidade Federal de Minas Gerais. Av. Antônio Carlos, 6627 - Pampulha - Belo Horizonte, Brazil. CEP 31270-901*

[b] *Centro de Desenvolvimento da Tecnologia Nuclear. Av. Antônio Carlos, 6627 - Pampulha - Belo Horizonte, Brazil. CEP 31270-901*

[c] *Department of Chemistry - Universidade Federal de Ouro Preto. Campus Morro do Cruzeiro, Rua Quatro, 786, Bauxita, Ouro Preto, Brazil. CEP: 35402-136*

[*] *fabianovargas@yahoo.com*

[*] *fabianovp@ufmg.br*

Electronic Supplementary Material

Calculation of desorption efficiency and reusability

The amount of Congo red adsorbed during cycle *n* was calculated from the difference between the initial dye concentration (C_0, mg L^{-1}) and the concentration measured after adsorption ($C_{ADS,n}$, mg L^{-1}):

$$w_{CR,ADS,n} = (C_0 - C_{ADS,n})V_{ADS} \tag{S1}$$

The corresponding adsorption capacity was calculated as:

$$q_{ADS,n} = \frac{(C_0 - C_{ADS,n})V_{ADS}}{m} \tag{S2}$$

where V_{ADS} (L) is the volume of the adsorption solution and m (g) is the mass of adsorbent. The amount (mg) of Congo red desorbed during cycle *n* was calculated according to:

$$w_{CR,DES,n} = C_{DES,n}V_{DES} \tag{S3}$$

where $C_{DES,n}$ (mg L^{-1}) is the concentration measured after desorption and V_{DES} (L) is the volume of the desorption solution. Because incomplete desorption may lead to progressive accumulation of Congo red on the adsorbent over successive cycles, a mass-balance correction was applied. The total mass of Congo red associated with the solid phase before desorption in cycle *n* was calculated as:

$$w_{CR,solid,n} = w_{CR,res,n-1} + w_{CR,ADS,n} \tag{S4}$$

where $w_{CR,res,n-1}$ (mg) is the residual Congo red mass remaining on the adsorbent after the previous cycle. For the first cycle, $w_{CR,res,0} = 0$.
The corrected desorption efficiency was determined as:

$$E_{DES,corr,n}/(\%) = \left(\frac{w_{CR,DES,n}}{w_{CR,solid,n}}\right) \times 100 \tag{S5}$$

The residual Congo red mass (mg) after desorption was calculated by:

$$w_{CR,res,n} = w_{CR,solid,n} - w_{CR,DES,n} \tag{S6}$$

Finally, the reusability efficiency was calculated as:

$$E_{reuse,n}/(\%) = \left(\frac{q_{ADS,n}}{q_{ADS,1}}\right) \times 100 \tag{S7}$$

where $q_{ADS,1}$(mg g^{-1}) is the adsorption capacity obtained in the first adsorption cycle.

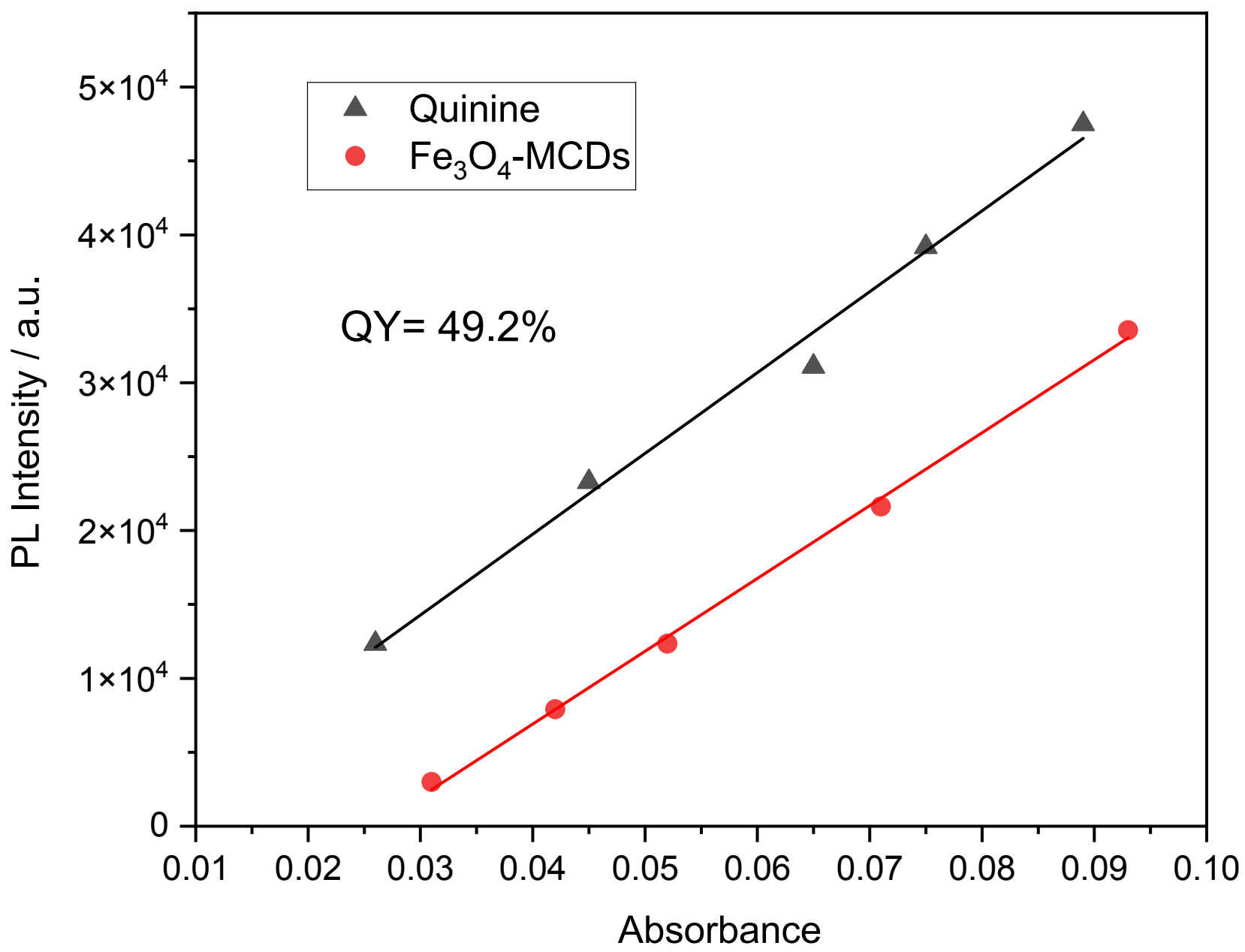

Figure S1: PL intensity and absorbance values of the standard quinine sulfate and Fe_3O_4-MCDs for fluorescence quantum yield determination.

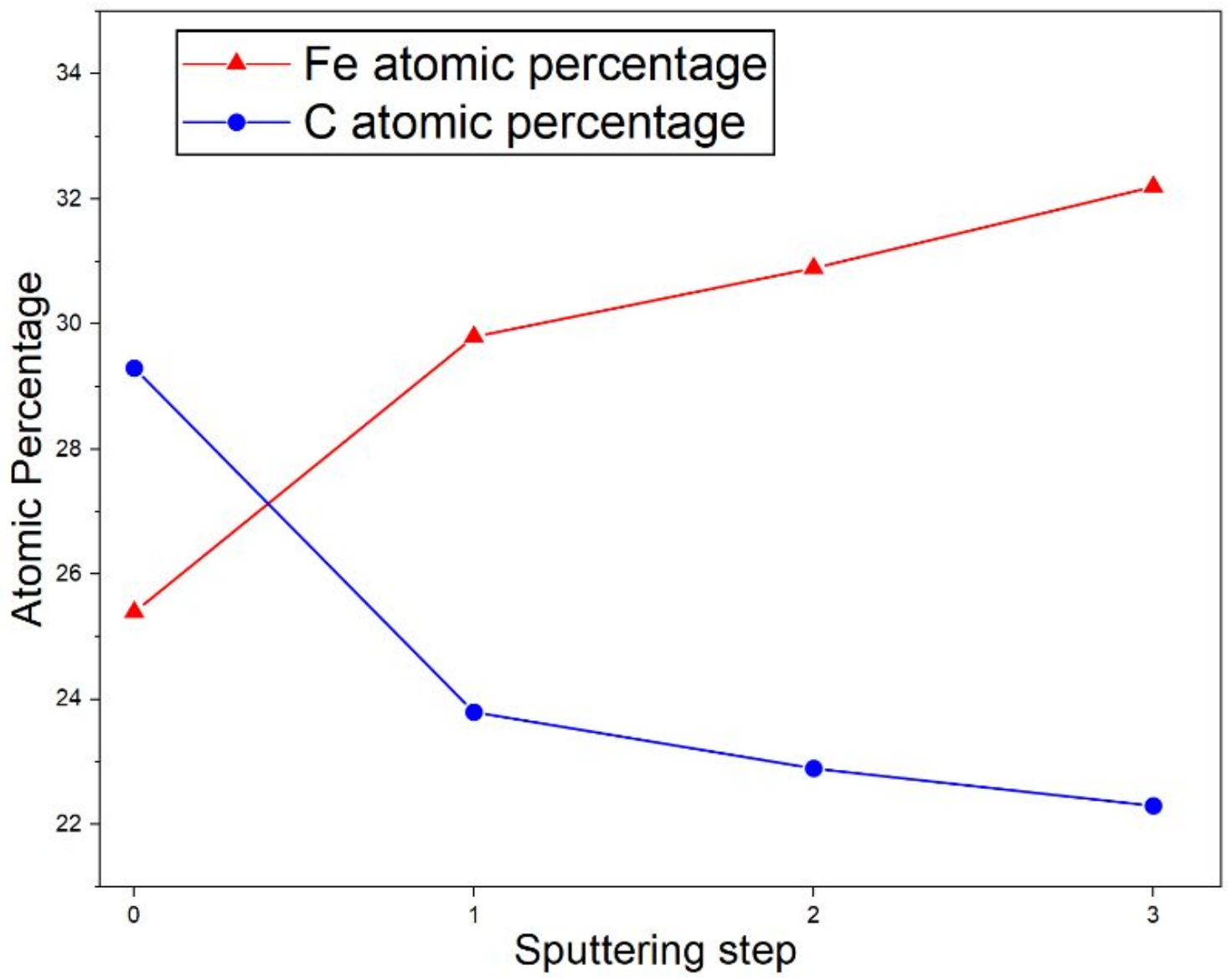

Figure S2: Evolution of the Fe and C atomic percentages during successive Ar^+ sputtering steps as obtained from XPS depth-profiling analysis.

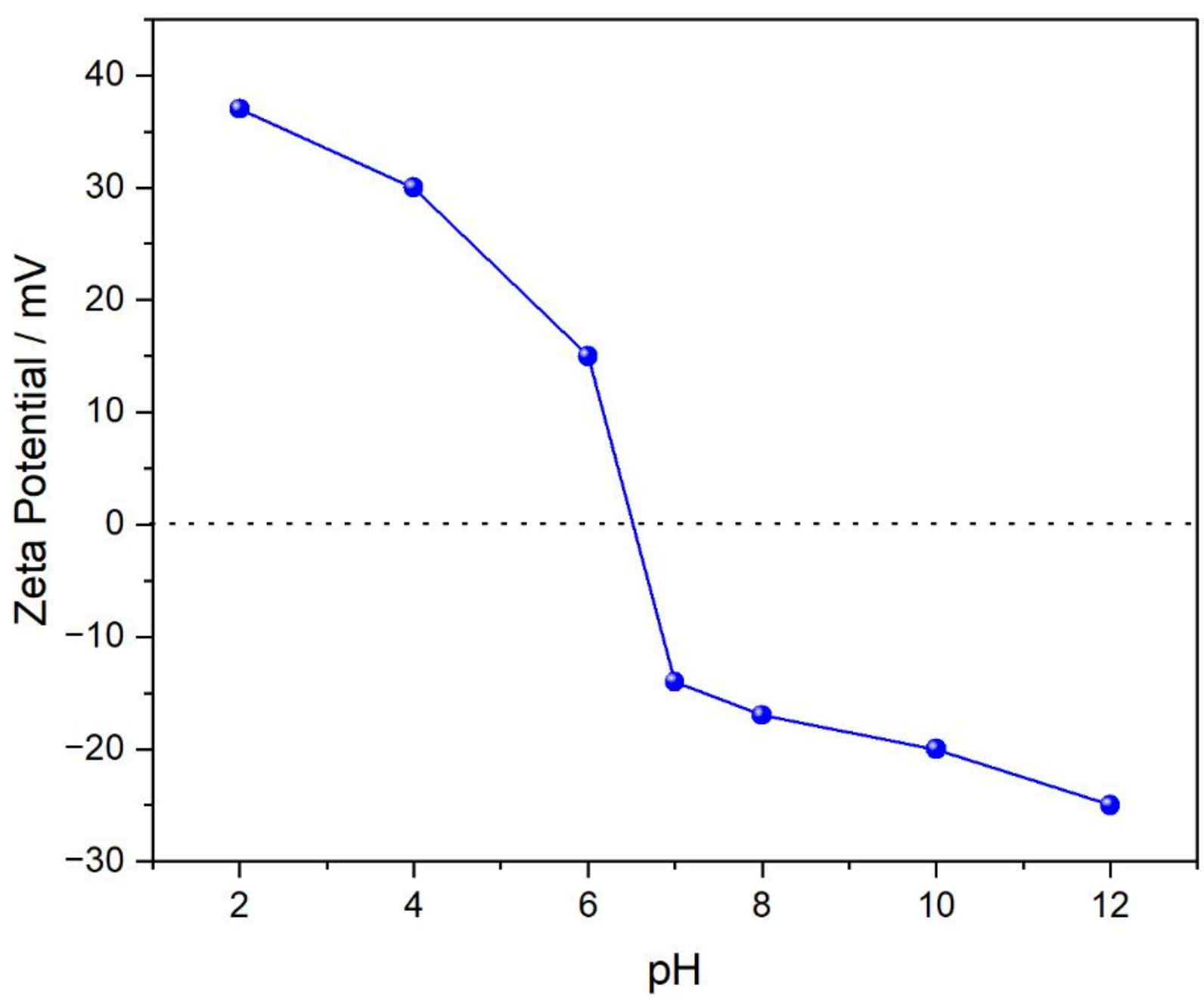

Figure S3: Zeta potential values of Fe_3O_4-MCDs as a function of solution pH.

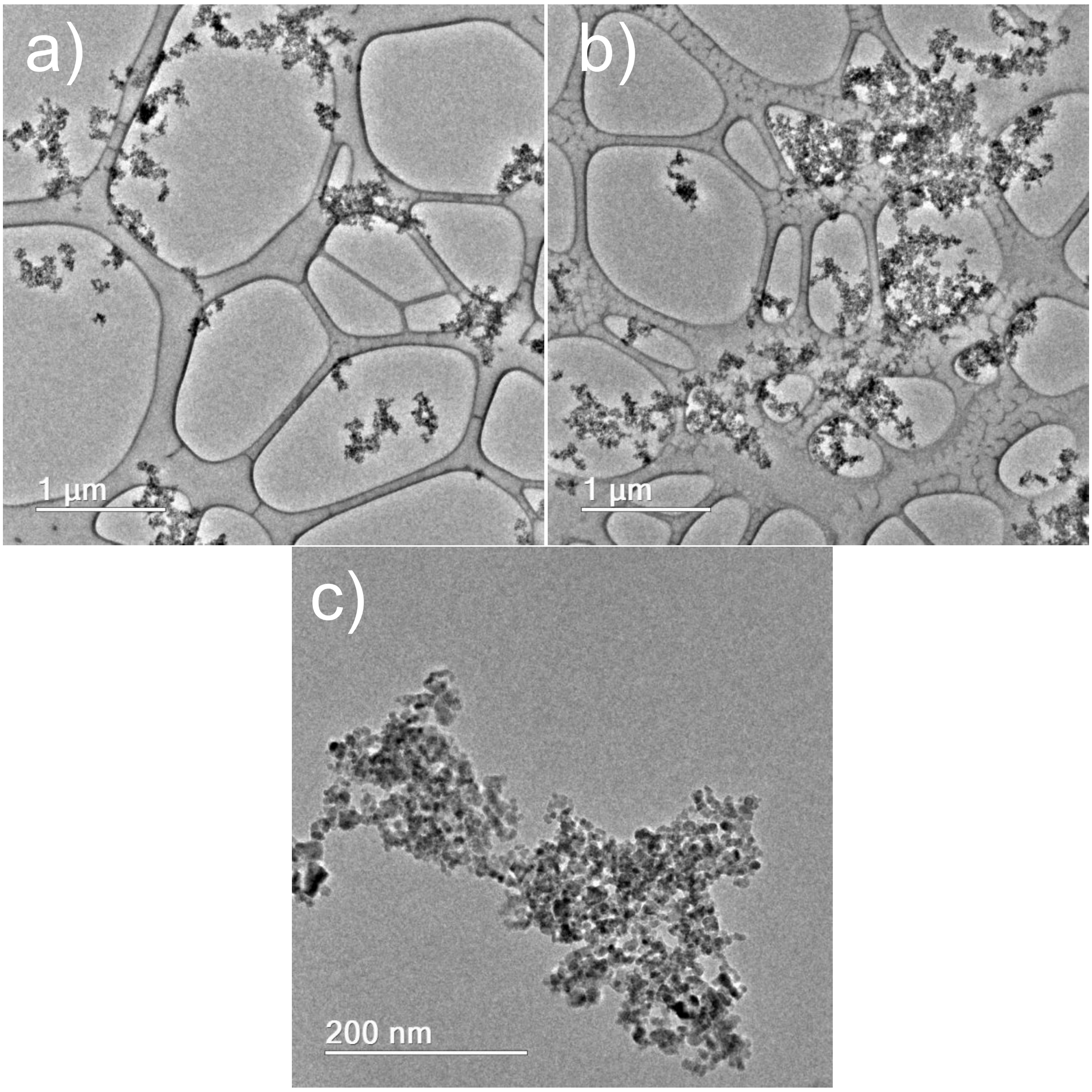

Figure S4: TEM images of the Fe_3O_4-MCDs acquired at low magnifications: (a) lowest magnification, (b) intermediate magnification, and (c) highest magnification.

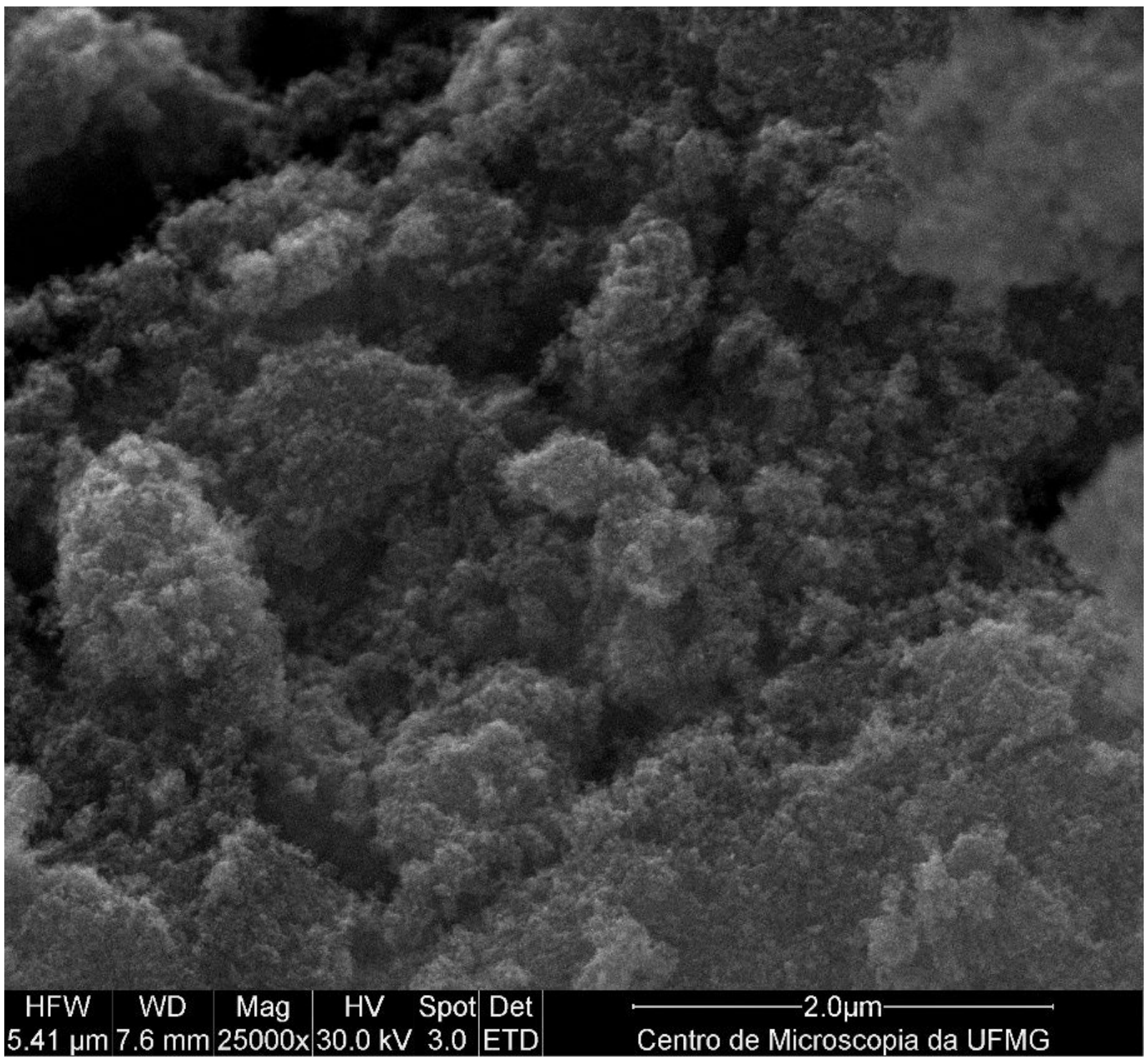

Figure S5: SEM image of Fe_3O_4-MCD in lower magnification.

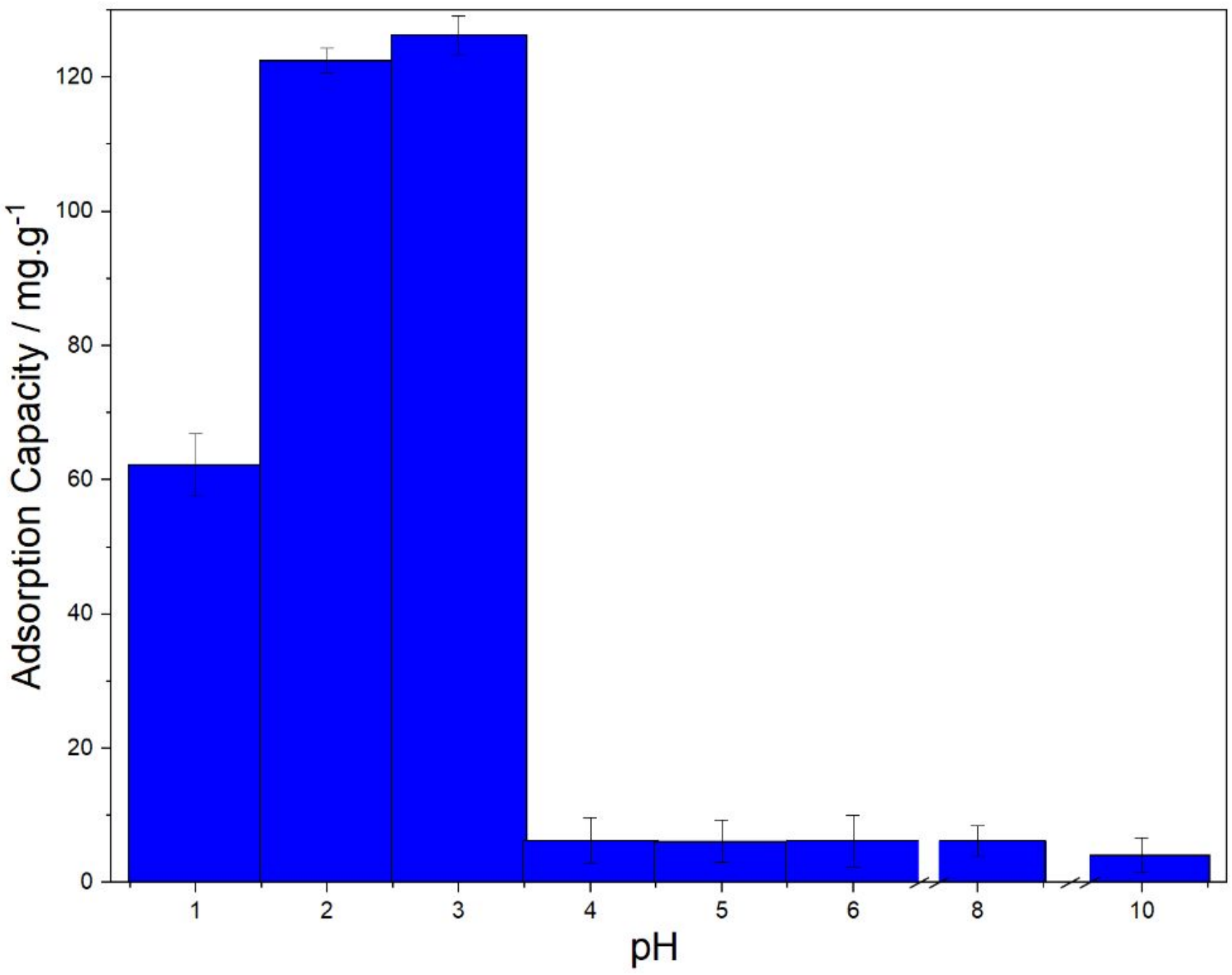

Figure S6: Effect of pH on the adsorption capacity of CR onto Fe_3O_4-MCDs. Error bars represent standard deviations from triplicate measurements.

Table S1: Kinetic and statistical parameters estimated from non-linear fitting of the experimental data with pseudo-first-order, pseudo-second-order, mixed-order, and intraparticle diffusion models at different initial concentrations.

Model	Parameter	40.00 mg L^{-1}	15.03 mg L^{-1}	5.65 mg L^{-1}	2.10 mg L^{-1}
**Experimental**	$q_{e,exp}$ / mg g^{-1}	128.8 ± 0.6	51.6 ± 0.5	18.5 ± 0.1	5.3 ± 0.4
	t_e / min	90	90	90	90
**Pseudo-first-order**	q_e / mg g^{-1}	122.3 ± 4.9	50.1 ± 1.3	18.0 ± 0.4	5.30 ± 0.4
	k_1 / min^{-1}	0.137 ± 0.022	0.211 ± 0.024	0.098 ± 0.008	0.06 ± 0.02
	R^2	0.9647	0.9829	0.9922	0.9290
	χ^2_{red}	89.91	6.808	0.498	0.536
**Pseudo-second-order**	q_e / mg g^{-1}	130.1 ± 2.3	52.6 ± 0.3	19.4 ± 0.6	5.88 ± 0.6
	k_2 / g mg^{-1} min^{-1}	0.0015 ± 0.002	0.0063 ± 0.0003	0.0064 ± 0.00097	0.011 ± 0.00627
	h / mg g^{-1} min^{-1}	25.4 ± 2.7	17.5 ± 0.7	2.43 ± 0.39	0.410 ± 0.22
	R^2	0.9946	0.9991	0.9896	0.9166
	χ^2_{red}	13.67	0.3393	0.6692	0.6293
**Mixed-order kinetics**	q_e /mg g^{-1}	127.1 ± 9.1	51.4 ± 0.5	18.1 ± 0.4	5.28 ± 0.46
	k_1 / min^{-1}	0.008 ± 0.045	0.020 ± 0.016	0.065 ± 0.032	0.059 ± 0.020
	k_2 / g mg^{-1} min^{-1}	0.0015 ± 0.0003	0.0060 ± 0.0005	0.0024 ± 0.0025	7.012E-15 ± 0.011
	R^2	0.9941	0.9991	0.9929	0.92895
	χ^2_{red}	17.89	0.4288	0.5502	0.64303
	f_1	0.04	0.06	0.60	1.00
	f_2	0.96	0.94	0.40	0.00
**Intraparticle diffusion model (3-segment PLR)**	C_{int} / mg g^{-1}	2.77 ± 3.99	—	—	—
	$K_{id},1$ / mg g^{-1} min$^{-0.5}$	26.14 ± 1.94	—	—	—
	$K_{id},2$ / mg g^{-1} min$^{-0.5}$	3.99 ± 1.59	—	—	—
	$K_{id},3$ / mg g^{-1} min$^{-0.5}$	0.43 ± 3.07	—	—	—
	J_1 / min$^{0.5}$	3.75 ± 0.37	—	—	—
	J_2 / min$^{0.5}$	10.31 ± 3.99	—	—	—
	t_1 / min	14.07 ± 2.75	—	—	—
	t_2 / min	106.39 ± 82.26	—	—	—
	q_1 / mg g^{-1}	100.80 ± 7.98	—	—	—
	q_2 / mg g^{-1}	127.02 ± 14.70	—	—	—
	R^2	0.99735	—	—	—
	χ^2_{red}	20.25983	—	—	—
**Intraparticle diffusion**	Cint / mg g^{-1}	—	1.50 ± 1.65	0.00 ± 1.37	0.00 ± 0.84

**model (2-segment PLR)**	K_{id},1 / mg g^{-1} $min^{-0.5}$	—	12.90 ± 0.80	3.59 ± 0.67	0.742 ± 0.274
	K_{id},2 / mg g^{-1} $min^{-0.5}$	—	0.300 ± 0.243	0.265 ± 0.201	0.000 ± 0.260
	J_1 / $min^{0.5}$	—	3.64 ± 0.21	4.60 ± 0.77	7.15 ± 2.86
	t_1 / min	—	13.24 ± 1.56	21.21 ± 7.09	51.16 ± 40.84
	q_1 / mg g^{-1}	—	48.45 ± 2.06	16.55 ± 1.62	5.31 ± 1.61
	R^2	—	0.99419	0.97524	0.88858
	χ^2_{red}	—	3.47746	2.38722	1.26014

Table S2: Isotherm and statistical parameters estimated from non-linear fitting of Langmuir, Freundlich, Sips, and Dubinin-Radushkevich models.

Model	Parameter	Parameter value
**Experimental**	$Q_{max,exp}$	121.6 ± 7.8
**Langmuir**	Q_{max} / mg g^{-1}	123.7 ± 10.6
	b / L mg^{-1}	0.196 ± 0.064
	R^2	0.8583
	RMSE / mg g^{-1}	16.87
**Freundlich**	K_F	37.9 ± 8.6
	n	3.36 ± 0.72
	R^2	0.7577
	RMSE / mg g^{-1}	22.06
**Sips**	Q_{max} / mg g^{-1}	121.3 ± 5.0
	b (L mg^{-1})	0.223 ± 0.024
	n	0.48 ± 0.09
	R^2	0.9545
	RMSE / mg g^{-1}	10.08
**Dubinin–Radushkevich**	q_m / mg g^{-1}	177.8 ± 19.3
	$B_{D\text{-}R}$ / mol^2 kJ^{-2}	0.00626 ± 0.00114
	E / kJ mol^{-1}	8.94 ± 0.81
	R^2	0.8283
	RMSE / mg g^{-1}	18.57

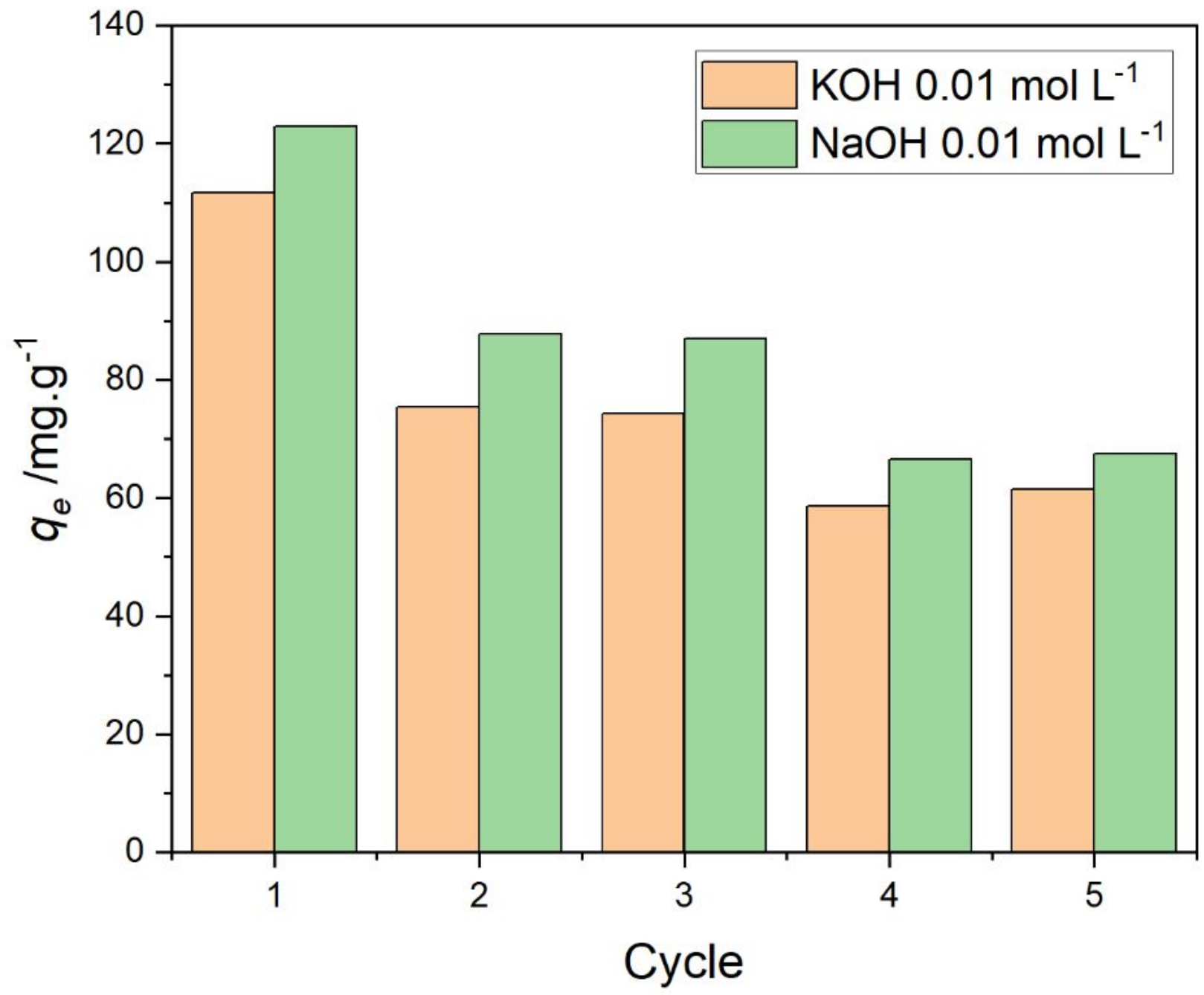

Figure S7. Adsorption capacity over successive regeneration cycles using 0.01 mol L^{-1} KOH and 0.01 mol L^{-1} NaOH as regeneration agents.

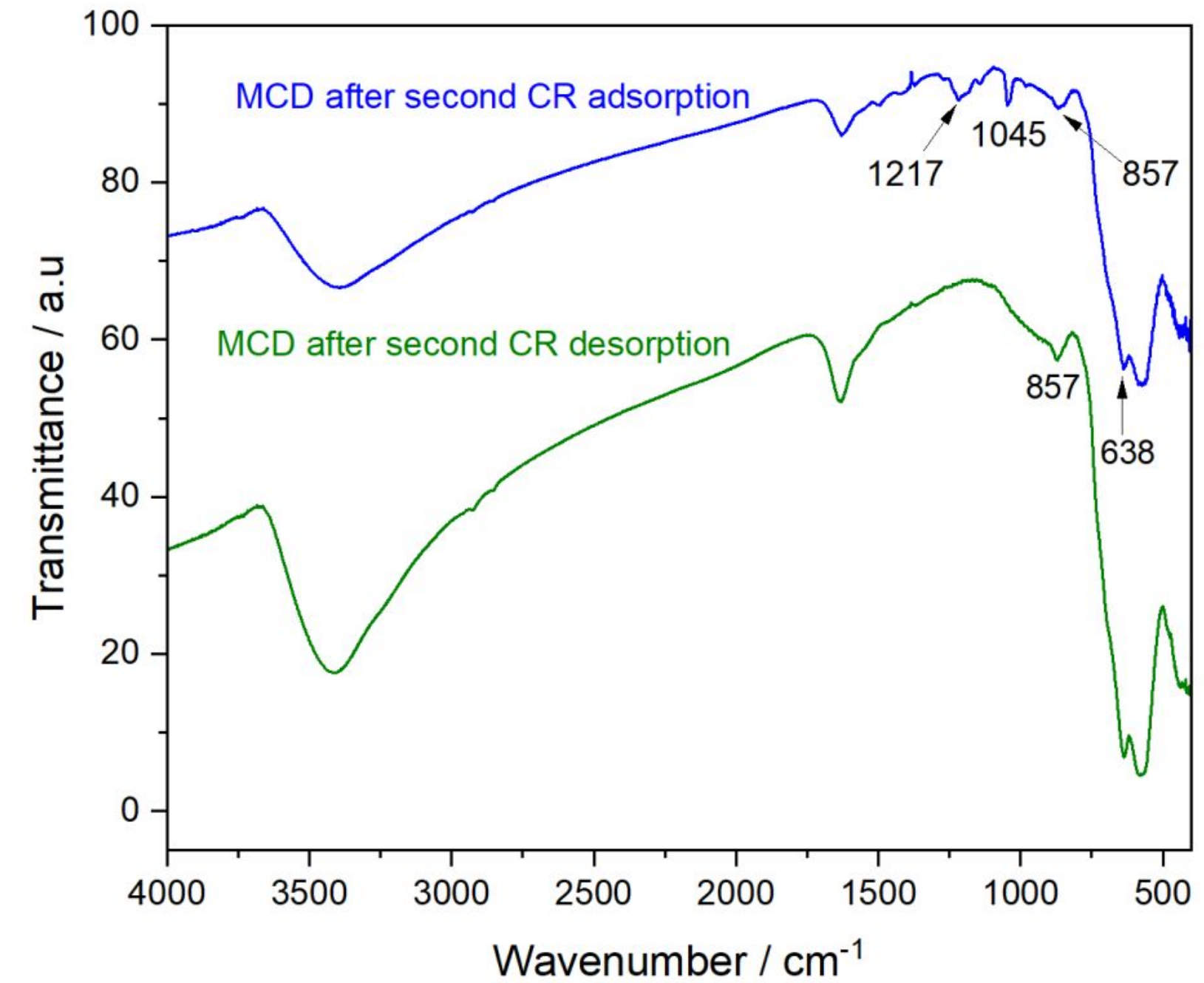

Figure S8: FTIR spectra of Fe_3O_4-MCD after the second CR adsorption and after the second dye desorption using 0.01 mol L^{-1} NaOH as the desorbing solution.

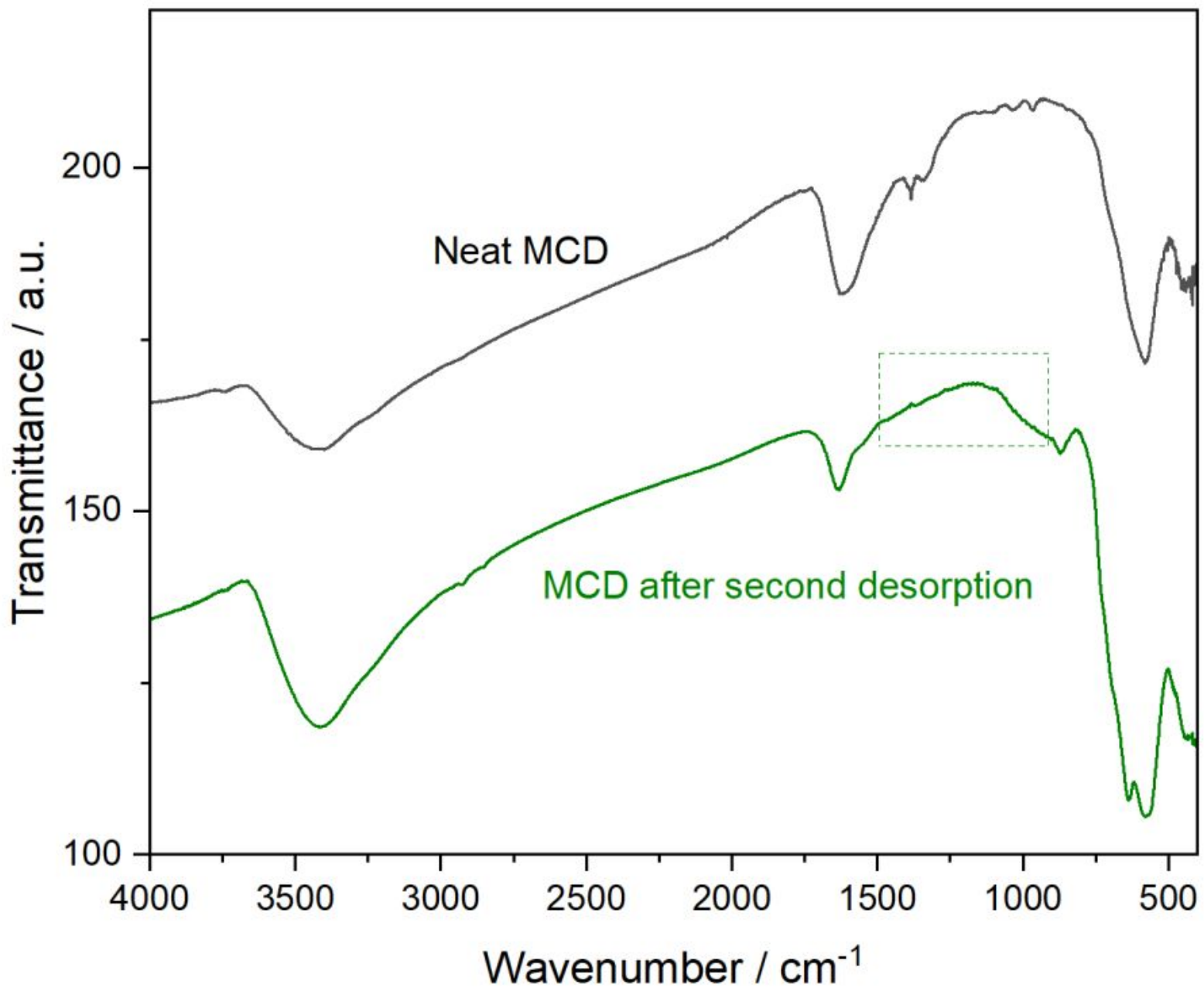

Figure S9: Comparison of the FTIR spectra of Fe_3O_4-MCD before adsorption (neat MCD) and after the second desorption cycle using 0.01 mol L^{-1} NaOH as the desorbing solution.
